# Protein loops are major contributors to DNA strand separation and high-fidelity substrate recognition for DNA methyltransferase CcrM

**DOI:** 10.1016/j.jbc.2026.111398

**Published:** 2026-03-24

**Authors:** Olivia Konttinen, Tyler Dangerfield, Jennifer Vargas, Kenneth A. Johnson, Norbert Reich

**Affiliations:** 1 Biomolecular Science and Engineering, University of California, Santa Barbara, Santa Barbara, California, USA; 2 Life Sciences Interdisciplinary Graduate Program, Department of Molecular Biosciences, University of Texas, Austin, Texas, USA; 3 Chemistry and Biochemistry, University of California, Santa Barbara, Santa Barbara, California, USA

**Keywords:** DNA strand separation, enzyme kinetics, global data fitting, DNA methylation, high-fidelity DNA recognition, stopped-flow, cell cycle–regulated DNA methyltransferase, CcrM

## Abstract

Strand separation is a newly described DNA recognition mechanism, and in the case of the cell cycle–regulated DNA methyltransferase (CcrM), it leads to an extraordinary level of substrate discrimination relative to other methyltransferases. The structural mechanisms underlying the process of DNA strand separation remain poorly understood. Two highly conserved loops in CcrM, loop-2B and loop-45, are inserted between the strand-separated DNA interface and are likely to generate and stabilize the strand-separated conformation. During strand separation, residues within loop-2B, loop-45, and loop-6E contact the DNA strand that undergoes methylation (target strand). Highly conserved loop residues R44 and F125 are positioned between the separated DNA strands and appear essential for maintaining the strand-separated intermediate. Replacement of F125 results in various perturbations of strand separation that are correlated to the size of the substituted residue. Global fitting of kinetic data shows that stabilization of DNA strand separation is perturbed by each mutation, leading to a reduced rate of methylation in some cases. These data support a functional role for these loops in generating and stabilizing the strand-separated intermediate. Insights into CcrM’s mechanism of DNA strand separation are likely applicable to understand strand-separation mechanisms for other enzymes.

Epigenetic tagging by DNA methylation is conserved across bacteria, humans, plants, and archaea (1, 2, 3, 4) and is responsible for transcriptional regulation, cell cycle regulation, DNA repair, DNA protection, tumor suppression, and several other important biological processes (5, 6, 7, 8). Aberrant bacterial DNA methylation can lead to loss of virulence and loss of protection against bacteriophages (9, 10, 11). Aberrant DNA methylation in humans leads to cancer, autoimmune diseases, metabolic disorders, and neurological disorders (12, 13). Thus, DNA methyltransferases are common drug targets for antibiotics and cancer therapeutics (14, 15, 16, 17).

Protein recognition of DNA sequences is fundamental to all organisms, and the underlying structural mechanisms are well understood (18). For example, zinc finger proteins contain a conserved structural motif using zinc to stabilize a DNA binding fold (19). Leucine zipper proteins contain two dimerized alpha helices, which contain basic amino acids at one terminus that recognize the major and minor DNA grooves (18). Helix–turn–helix proteins contain a structural motif comprised of two alpha helices connected by a short flexible peptide in which the recognition helix is inserted within the DNA major groove where protein residues participate in base-specific DNA recognition (20, 21, 22). These and other motifs are found in diverse proteins that recognize a vast variety of DNA sequences (23).

DNA methyltransferases recognize specific sequences and modify adenines or cytosines (1, 8). The majority of these bacterial and eukaryotic enzymes rely on a “base flipping” mechanism in which the target base is flipped out of the DNA double helix. In the case of the well-studied M.HhaI C^5^-cytosine MTase, a two-loop DNA-binding motif is used for both sequence recognition and base flipping (24, 25, 26, 27). Movement of one of these loops is coupled with base flipping (24, 25, 26, 27). The homotetrameric human DNMT3A is a C^5^-cytosine DNA methyltransferase and relies on a target recognition domain (TRD), which is made up of a loop that makes major groove contacts to the recognition sequence CpG (28, 29). The TRD loop is inserted into the DNA major groove, where it makes specific contacts (28). The TRD loop’s flexibility has also been proposed *via* molecular dynamic simulations to be important for DNMT3A recognition and catalysis (30).

The bacterial N^6^-adenine DNA methyltransferase CcrM (cell cycle–regulated DNA methyltransferase) dimer methylates adenine in the sequence 5′GANTC3′ and is the first DNA methyltransferase shown to rely on a unique DNA recognition mechanism in which the DNA strands are separated, and most recognition interactions are limited to only one strand (target) (31).

Loop-2B and loop-45 are inserted between the DNA strands, making contacts with target-strand bases, and appear to be stabilized by interactions with other protein moieties (32, Fig. 1). Four of the five base pairs within the recognition site become disrupted as loop-2B approaches DNA from the major groove and makes base-specific interactions with the target strand (32, Fig. 1). Loop-45 approaches DNA from the minor groove and is positioned between the DNA strands. The early biochemical evidence that CcrM readily methylates single-stranded DNA with similar fidelity supports the structural model (32).

**Figure 1:**
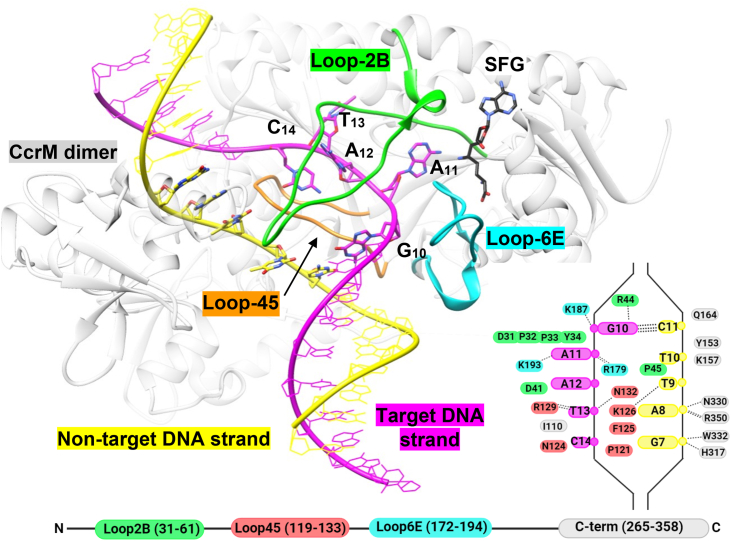
**Cocrystal structure of CcrM complexed with dsDNA and sinefungin (SFG).** Loop-2B (*green*) and loop-45 (*orange*) are positioned within the separated interface of DNA. Loop-6E (*cyan*) is positioned near the target adenine and methyl-donor cofactor SFG. The GANTC recognition site is annotated 5′-G_10_A_11_A_12_T_13_C_14_-3’. The structural image was made with UCSF Chimera from Protein Data Bank: 6PBD (31). *Inset,* the strand-separated DNA phosphate backbone is depicted by the *black outline*. The DNA phosphates are depicted as *circles* and DNA bases as *ovals*. Amino acids that interact with nucleic acids are depicted as *ovals*. Hydrogen bonds are *dashed black lines*. *Bottom,* the 2D positioning of loops within the CcrM N-terminal domain relative to the C terminus. The *cartoon images* were made with BioRender. CcrM, cell cycle–regulated DNA methyltransferase.

The target strand recognition sequence (G_10_A_11_A_12_T_13_C_14_) is structurally perturbed in the strand-separated state (Fig. 2*A*). A_11_, T_13_, and C_14_ are flipped outside the DNA helix (Fig. 2*A*). G_10_ maintains Watson–Crick base pairing to C_11_ of the nontarget strand, and G_10_ is recognized by R44 from loop-2B, which is a highly conserved residue necessary for catalysis. G_10_ also makes a hydrogen bond to the peptide backbone of loop-45 (Fig. 2*B*). A_11_ (the target adenine) is positioned for methyl transfer, and the conserved catalytic DPPY-motif (D31, P32, P33, and Y34) within loop-2B makes hydrogen bonds and stacking interactions with A_11_. K193 from loop-6E makes a hydrogen bond to A_11_, likely contributing to base-specific recognition and stabilization. R179 also hydrogen bonds to the phosphate backbone of A_11_ (Fig. 2*C*). A_12_ is not recognized by a protein residue, which is not surprising because of CcrM’s ability to accommodate any base at this N-position (5′GANTC3′) (Fig. 2*D*). T_13_ is recognized by R129 from loop-45 *via* hydrogen bonds and W109 *via* stacking (Fig. 2*E*). C_14_ makes three hydrogen bonds to the peptide backbone of loop-45. Last, C_14_ is recognized by N124 from loop-45 *via* stacking interactions (Fig. 2*F*).

**Figure 2:**
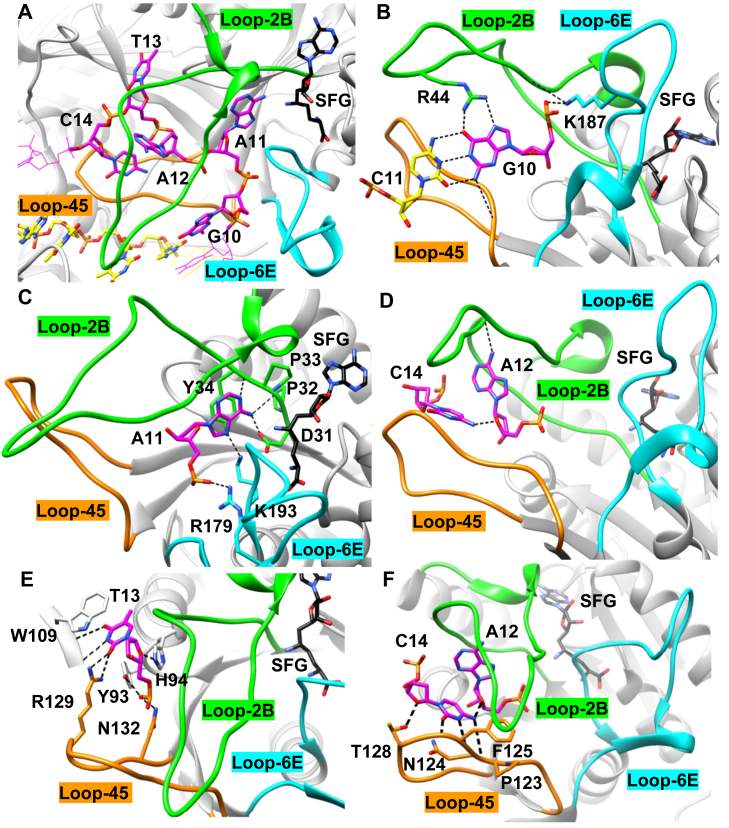
**Base-specific recognition by loop-405, loop-2B, and loop-6E.***A*, the target strand recognition sequence (G_10_A_11_A_12_T_13_C_14_) is strand separated with loop-45 (*orange*) and loop-2B (*green*) positioned between the DNA strands. Loop-6E (*cyan*) is near the target adenine and methyl-donor cofactor sinefungin (SFG). *B*, G_10_ maintains Watson–Crick base pairing to C_11_ of the nontarget strand. G_10_ is recognized by R44 from loop-2B, which is a highly conserved residue necessary for catalysis. K187 makes a hydrogen bond to the phosphate backbone of G_10_. G_10_ also makes a hydrogen bond to the peptide backbone of loop-45. *C*, A_11_ (the target adenine) is positioned for methyl transfer. The DPPY-motif (D31, P32, P33, and Y34) within loop-2B makes hydrogen bonds and stacking interactions with A_11_. K193 from loop-6E makes a hydrogen bond to A_11_, likely contributing to base-specific recognition and stabilization. R179 makes a hydrogen bond to the phosphate backbone of A_11_. *D*, A_12_ is not recognized by a protein residue, which is not surprising because of the ability of CcrM to accommodate any base at this N-position (5′GANTC3′). *E*, T_13_ is recognized by R129 from loop-45 *via* hydrogen bonds and W109 *via* stacking. *F*, C_14_ makes three hydrogen bonds to the peptide backbone of loop-45. C14 also makes a stacking interaction to N124. The structural image was made with UCSF Chimera from the Protein Data Bank: 6PBD (31). CcrM, cell cycle–regulated DNA methyltransferase.

We previously showed that CcrM’s highly conserved and unusual 83 amino acid C terminus is essential for DNA binding and strand separation (33) and that DNA strand separation is tightly coupled to substrate discrimination (34). Global fitting resolved a kinetic model in which CcrM binds DNA, and the protein undergoes a conformational change, followed by DNA strand separation and methylation (34). However, the mechanistic details that lead up to DNA strand separation, including the role of loops that stabilize the strand-separated state, have not been defined. In this work, we examined mutations of residues in the two highly conserved loops (loop-2B and loop-45) using stopped-flow fluorescent kinetic methods and employing global fitting to assess how loop-2B and loop-45 govern strand separation to facilitate substrate discrimination and catalysis.

## Results

### Loop-2B and loop-45 are positioned within the strand-separated DNA and make base-specific contacts to the target strand recognition sequence

The CcrM cocrystal structure reveals that loop-2B, loop-45, and loop-6E are implicated in DNA strand separation and base recognition (Fig. 1, 32). Loop-2B and loop-45 are positioned within the strand-separated DNA interface and make base-specific contacts with the target-strand bases, whereas loop-6E is positioned outside the strand-separated interface and interacts with target-strand bases in extrahelical positions (Figs. 1 and 2). We wanted to understand how these closely packed loops contribute to generating or stabilizing DNA strand separation, or both.

### Simplified WT CcrM kinetic model

**Figure 3:**
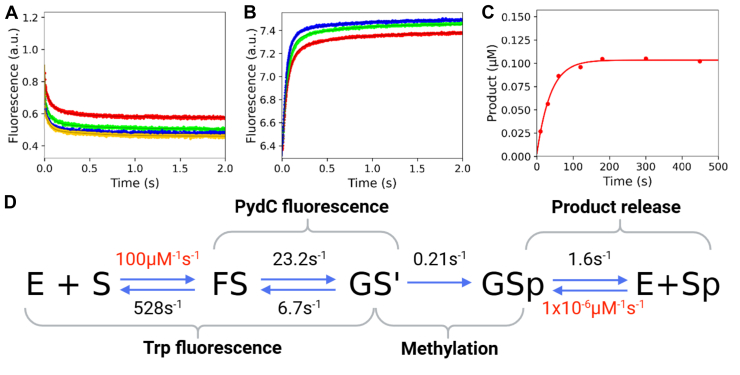
**WT CcrM enzyme kinetic model shows that DNA strand separation is the rate-limiting step in catalysis.***A*, Trp fluorescence monitored the kinetics of binding and protein isomerization and consisted of WT CcrM (500 nM), C1-DNA (2.5 [*red*], 5.0 [*green*], 7.5 [*blue*], and 10.0 [*yellow*] μM), and SAM (60 μM). *B*, DNA strand–separation kinetics monitored by PydC fluorescence consisted of P1-DNA (1.0 μM), WT CcrM (5.0 [*red*], 7.5 [*green*], and 10.0 [*blue*] μM), and SAM (60 μM). *C*, single-turnover methylation monitored *via* radiochemical methods consisted of C1-DNA (100 nM), WT CcrM (250 nM), and tritiated SAM (15 μM). *D*, global fitting of the data defining the kinetic steps and rate constants for WT CcrM and dsDNA. All experiments (*A*–*C*) were globally fit in KinTek Explorer, and *solid lines* represent the simulated traces from the global fit. Rates in *red* were locked during the simulation, whereas rates in *black* were allowed to float. CcrM, cell cycle–regulated DNA methyltransferase.

We previously reported a five-step kinetic model for CcrM with rate constants derived from global fitting (34). We showed that Trp fluorescence monitored enzyme–DNA association and a subsequent protein conformational change (k_1_, k_-1_, k_2_, and k_-2_), PydC fluorescence monitored DNA strand separation (k_2_, k_-2_, k_3_, and k_-3_), where the second phase of Trp kinetics is kinetically coupled to the first phase of PydC kinetics, and a radiochemical methylation assay monitored product formation (k_4_) (34). We have simplified our WT model to four steps by removing the GS^II^ intermediate (Fig. 3). This four-step model increases simplicity, and each rate is kinetically defined by our data. The Trp and PydC kinetics (Fig. 3, *A* and *B*, respectively) are identical to our previous five-step model (34). In this work, we used unlabeled DNA in the methylation experiment (Fig. 3*C*) compared with PydC-labeled DNA in the methylation experiment in our previous five-step model (34). PydC–DNA was utilized previously in the methylation experiment to have consistency between the PydC-fluorescence stopped-flow assay monitoring DNA strand separation and the radiochemical assay monitoring methylation (34). Here, unlabeled DNA was used in the methylation experiment for WT and all mutants because the PydC-labeled DNA had too low of a signal for the mutants, whereas unlabeled DNA had a substantial radiochemical signal. The resulting kinetic model shows that methylation (*k*_3_) is rate limiting (Fig. 3*D*). To explore the structural basis for our previously published CcrM kinetic model (34), we collected kinetic data for loop mutants in loop-2B and loop-45 to understand how these loops regulate DNA strand separation and catalysis. Residues selected for mutation are highly conserved and are structurally implicated in generating and/or stabilizing DNA strand separation.

### Loop residue F125 size contributes to generation and stabilization of GS^I^

**Figure 4:**
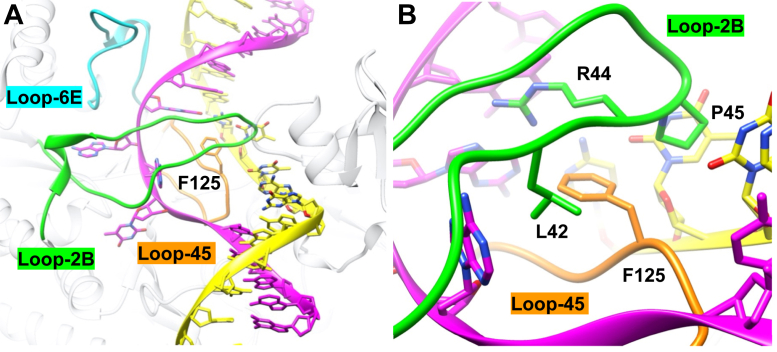
**F125 from loop-45 is positioned between the strand-separated DNA and does not make base-specific contacts with the nucleic acids of the recognition site.***A*, loop-45 (*orange*) approaches the strand-separated DNA from the minor groove, and loop-2B (*green*) approaches the strand-separated DNA from the major groove. Loop-6E (*cyan*) is shown for reference. F125 protrudes out of loop-45 and is positioned between the DNA strands. *B*, a closer view of F125 protruding between the strand-separated interface. The structural image was made with UCSF Chimera from the Protein Data Bank: 6PBD (31).

F125 in loop-45 was selected for mutation because of its positioning within the DNA strand–separated bubble. F125 does not make any base-specific interactions and is therefore unlikely to be directly involved in base-specific recognition (Fig. 4); however, it does make hydrophobic interactions with L42 in loop-2B, acting as a pin to keep the strands separated. We predicted that the size of F125 could contribute to either generation of DNA strand separation, stabilization of DNA strand separation, or both through interactions with loop-2B. We therefore mutated F125 to either leucine, alanine, or tryptophan to probe size at this position.

**Figure 5:**
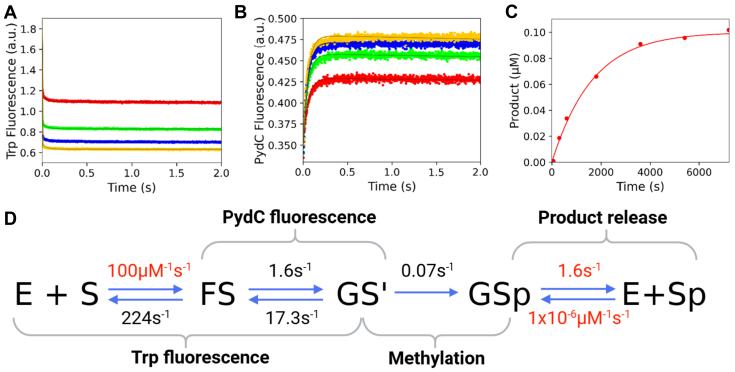
**F125L destabilizes the GS^I^ state, decreasing *k*cat.***A*, F125L can bind DNA, and the protein isomerization event occurs as seen with WT. Trp fluorescence monitored the kinetics of binding and protein isomerization and consisted of F125L (500 nM), C1-DNA (2.5 [*red*], 5.0 [*green]*, 7.5 [*blue*], and 10.0 [*yellow*] μM), and SAM (60 μM). *B*, F125L can strand separate DNA. However, the strand-separated state (GS^I^) is destabilized. DNA strand–separation kinetics monitored by PydC fluorescence consisted of P1-DNA (1.0 μM), F125L (2.5 [*red*], 5.0 [*green*], 7.5 [*blue*], and 10.0 [*yellow*] μM), and SAM (60 μM). *C*, F125L can methylate DNA but at a slower observed rate than WT. Single-turnover methylation monitored *via* radiochemical methods consisted of C1-DNA (100 nM), F125L (250 nM), and tritiated SAM (15 μM). *D*, global fitting of the data for F125L shows that *K*_*2*_ is now unfavorable because of destabilization of GS^I^. All experiments (*A*–*C*) were globally fit in KinTek Explorer. *Solid lines* represent the simulated traces from the global fit. Product release was not defined for F125L; therefore, *k*_4_ was locked at the WT value (1.63 s^−1^). Rates in *red* were locked during the simulation, whereas rates in *black* were allowed to float.

We first characterized the F125L mutant. F125L binds DNA with a similar affinity to WT in equilibrium DNA-binding measurements; F125L *K*_*d*_^app^ = 185 ± 19 nM, WT *K*_*d*_^app^ = 150 ± 5 nM (Fig. S1) (34). We next looked at the kinetics of strand separation and methylation with the stopped-flow and radiochemical assays described before. From the stopped-flow experiments, the formation of the GS^I^ intermediate was perturbed by lowering *k*_*2*_ by a factor of ∼15 (from 23.2 s^−1^ to 1.6 s^−1^) and increasing the rate of DNA annealing by a factor of 2 (from 6.7 s^−1^ to 17.3 s^−1^), resulting in a net change of the equilibrium constant for this step from ∼3.5 to ∼0.09, a 39-fold decrease in the stability of the strand-separated GS^I^ intermediate. Global fitting showed that the rate of methylation was only slightly perturbed, at 0.07 s^−1^ relative to 0.21 for WT CcrM (Fig. 5, *A*–*C*, Table 1). The rate constants for this mutant are given in the kinetic scheme in Figure 5*D*. The hydrophobic interaction that F125 makes with loop-2B is stronger than with F125L, resulting in decreased kinetic parameters (*k*_cat_, *K*_*m*_, and *k*_cat_*/K*_*m*_) for F125L (Table 2). Confidence contour analysis for F125L shows that the kinetic parameters are well defined by the data (Fig. S2). While our kinetic models for each mutant have product release (*k*_4_) modeled as a discrete step, global data fitting showed that this rate constant is not defined by the data, as it is much faster than the methylation step, so we locked this parameter at the WT value (1.63 s^−1^) for all mutants discussed in this article.

**Table 2:** *k*_cat_, *K*_*m*_, *k*_cat_/*K*_*m*_

Enzyme	*k*_cat_ (s^−1^)	*K*_*m*_ (μM)	*k*_cat_/*K*_*m*_ (μM^−1^ s^−1^)
WT	0.15 ± 0.05	1.1 ± 0.04	0.13 ± 0.05
F125L	0.006 ± 8.2 × 10^−4^	2.1 ± 0.02	0.003 ± 4.0 × 10^−4^
F125A	2.0 × 10^−4^ ± 5.4 × 10^−5^	5.9 ± 0.13	3.4 × 10^−5^ ± 9.1 × 10^−6^
F125W	0.01 ± 7.4 × 10^−4^	1.1 ± 0.04	0.01 ± 0.001
R129A	3.5 × 10^−5^ ± 6.4 × 10^−6^	1.4 ± 0.04	2.5 × 10^−5^ ± 4.7 × 10^−6^
N124A	2.7 × 10^−4^ ± 3.2 × 10^−5^	1.7 ± 0.05	1.6 × 10^−4^ ± 2.0 × 10^−5^
R44A	5.0 × 10^−5^ ± 3.9 × 10^−6^	1.8 ± 0.06	2.7 × 10^−5^ ± 2.3 × 10^−6^

Steady-state parameters were calculated from the rate constants in Table 1 using the equations listed in the Experimental procedures section.

**Figure 6:**
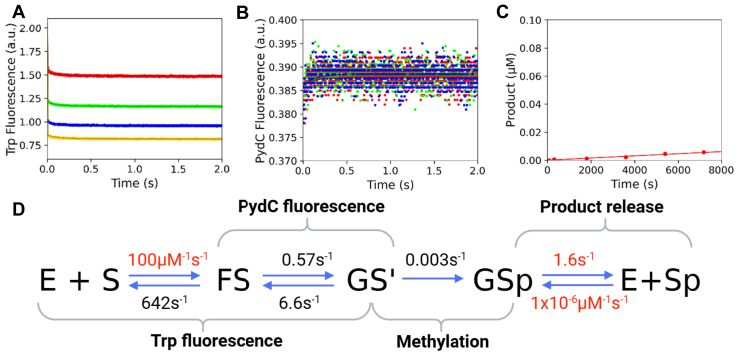
**F125A destabilizes FS and GS^I^, resulting in a lack of methylation.***A*, F125A can bind DNA and undergo protein isomerization; however, the protein–DNA complex (FS) is destabilized. Trp fluorescence monitored the kinetics of binding and protein isomerization and consisted of F125A (500 nM), C1-DNA (5.0 [*red*], 7.5 [*green*], and 10.0 [*yellow*] μM, and SAM (60 μM). *B*, F125A cannot strand-separate DNA, or the GS^I^ state is so destabilized that it precludes detection by PydC fluorescence. DNA strand–separation kinetics monitored by PydC fluorescence consisted of P1-DNA (1.0 μM), F125A CcrM (2.5 [*red*], 5.0 [*green*], 7.5 [*blue*], and 10.0 [*yellow*] μM], and SAM (60 μM). *C*, F125A is greatly hindered in DNA methylation, as seen by the very small amount of product formation. Single-turnover methylation monitored *via* radiochemical methods consisted of C1-DNA (100 nM), F125A (250 nM), and tritiated SAM (15 μM). *D*, global fitting of the data for F125A shows that *K*_2_ is now unfavorable because of destabilization of GS^I^. All experiments (*A*–*C*) were globally fit in KinTek Explorer. Product release was not defined for F125A; therefore, *k*_4_ was locked at the WT value (1.63 s^−1^). Rates in *red* were locked during the simulation, whereas rates in *black* were allowed to float. *Solid lines* represent the simulated traces from the global fit. CcrM, cell cycle–regulated DNA methyltransferase.

Next, we sought to probe the role of F125 further with an even smaller amino acid replacement (F125A). Equilibrium measurements show that the F125A mutant binds DNA with a similar *K*_*d*_^app^ compared with WT; F125A *K*_*d*_^app^ = 190 ± 30, WT *K*_*d*_^app^ = 150 ± 5 nM (Fig. S1). Kinetic experiments show that the F125A mutation destabilized GS^I^ to a greater extent than F125L (F125A *k*_2_ = 0.6 s^−1^ and *k*_*-*2_ = 6.6 s^−1^, Fig. 6, *A* and *B*, Table 1). The GS^I^ state is so destabilized that there was no detectable PydC fluorescence signal with this mutant (Fig. 6*B*, Table 1). F125A showed a rate of methylation reduced by approximately two orders of magnitude; F125A *k*_3_ = 0.003 s^−1^, WT *k*_3_ = 0.21 s^−1^). The resulting product formation for F125A was very slow on the time scale of the experiment (Fig. 6*C*). Again, product release (*k*_4_) was locked at the WT value (1.63 s^−1^). Confidence contour analysis for F125A shows that the kinetic parameters are well defined by the data (Fig. S2). The combination of decreased strand separation and methylation suggests that size at position 125 not only affects strand separation but is important in the alignment of catalytic residues for methylation, as demonstrated by the drop in *k*_cat_ for these mutants.

**Figure 7:**
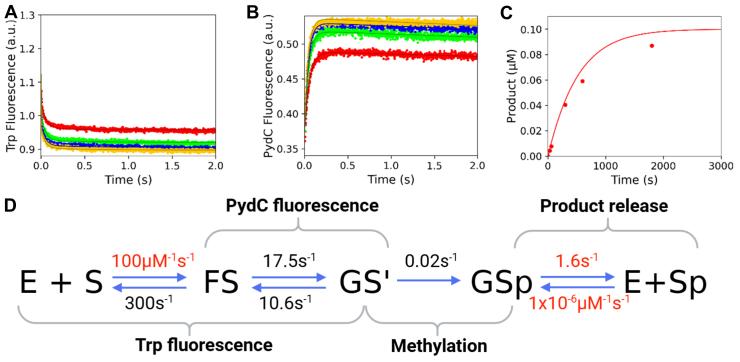
**F125W has similar Trp and PydC kinetics, whereas methylation is reduced relative to WT.***A*, F125W can bind DNA, and the protein isomerization event occurs as seen with WT. Trp fluorescence monitored the kinetics of binding and protein isomerization and consisted of F125W (500 nM), C1-DNA (2.5 [*red*], 5.0 [*green*], 7.5 [*blue*], and 10.0 [*yellow*] μM), and SAM (60 μM). *B*, F125W can strand-separate DNA, and the resulting GS^I^ state is comparably stable relative to WT. DNA strand–separation kinetics monitored by PydC fluorescence consisted of P1-DNA (1.0 μM), F125W (2.5 [*red*], 5.0 [*green*], 7.5 [*blue*], and 10.0 [*yellow*] μM), and SAM (60 μM). *C*, F125W can methylate DNA but at a slower observed rate than WT. Single-turnover methylation monitored *via* radiochemical methods consisted of C1-DNA (100 nM), F125W (250 nM), and tritiated SAM (15 μM). *D*, global fitting of the data for F125W shows that methylation is reduced relative to WT, whereas the preceding states (FS and GS^I^) are stable. All experiments (*A*–*C*) were globally fit in KinTek Explorer. Product release was not defined for F125W; therefore, *k*_4_ was locked at the WT value (1.63 s^−1^). Rates in *red* were locked during the simulation, whereas rates in *black* were allowed to float. *Solid lines* represent the simulated traces from the global fit.

We next introduced greater size at position 125 with the F125W mutant. Equilibrium binding measurements show that this mutant also binds DNA with similar affinity to WT; F125W *K*_*d*_^app^ = 136 ± 16, WT *K*_*d*_^app^ = 150 ± 5 nM (Fig. S1). Stopped-flow kinetics, however, show that F125W undergoes the initial protein conformational and strand separation slightly faster than WT; F125W *k*_2_ = 17.5 s^−1^ and *k*_*-*2_ = 10.6 s^−1^, WT *k*_2_ = 23.2 s^−1^ and *k*_*-*2_ = 6.7 s^−1^ (Fig. 7, *A* and *B*, Table 1). F125W methylates with a 12-fold reduction from WT; F125W *k*_3_ = 0.02 s^−1^ (Fig. 7*C*, Table 1). Global fitting of the data for F125W shows that the precatalytic states FS and GS^I^ are stabilized while methylation is decreased relative to WT (Fig. 7*D*). Confidence contour analysis for F125W shows that the kinetic parameters are well defined by the data (Fig. S2).

### Loop residues that interact with specific recognition site bases are responsible for generation and stabilization of GS^I^

**Figure 8:**
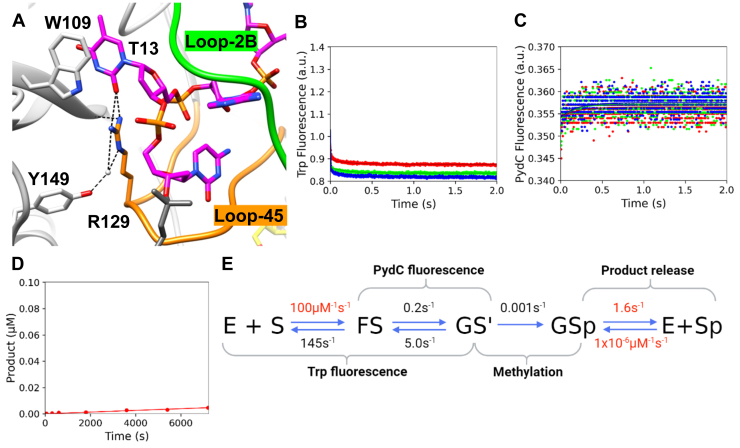
**The R129A mutant is greatly hindered in forming and stabilizing the strand-separated intermediate (GS^I^), resulting in lack of methylation.***A*, R129 from loop-45 makes two hydrogen bonds to T_13_ of the target strand recognition site (5′GANTC3′). *B*, R129A can bind DNA more tightly than WT, stabilizing the FS state. Trp fluorescence monitored the kinetics of binding and protein isomerization and consisted of R129A (500 nM), C1-DNA (2.5 [*red*], 5.0 *[green*], and 7.5 [*blue*] μM), and SAM (60 μM). *C*, R129A cannot strand-separate DNA, or the GS^I^ state is so destabilized that it precludes detection by PydC fluorescence. DNA strand–separation kinetics monitored by PydC fluorescence consisted of P1-DNA (1.0 μM), R129A (2.5 [*red*], 5.0 [*green*], and 7.5 [*blue*] μM), and SAM (60 μM). *D*, R129A very slowly methylates DNA, as seen by the small amount of product formation on the time scale of the experiment. Single-turnover methylation monitored *via* radiochemical methods consisted of C1-DNA (100 nM), R129A (250 nM), and tritiated SAM (15 μM). *E*, global fitting of the data for R129A shows that *K*_2_ is now unfavorable because of destabilization of GS^I^. All experiments (*A*–*C*) were globally fit in KinTek Explorer. Product release was not defined for R129A; therefore, *k*_*4*_ was locked at the WT value (1.63 s^−1^). Rates in *red* were locked during the simulation, whereas rates in *black* were allowed to float. *Solid lines* represent the simulated traces from the global fit.

R129 is a highly conserved residue from loop-45 that makes two hydrogen bonds to T_13_ within the target strand recognition site, which stacks with W109 (Fig. 8*A*) and also forms an additional hydrogen bond with Y149. R129A binds DNA with a similar affinity to WT; R129A *K*_*d*_^app^ = 135 ± 7 nM, WT *K*_*d*_^app^ = 150 ± 5 nM (Fig. S1). Kinetics experiments show that the R129A GS^I^ strand–separated intermediate is perturbed approximately 90-fold; R129A *k*_2_ = 0.2 s^−1^ and *k*_*-*2_ = 5.0 s^−1^, WT *k*_2_ = 23.2 s^−1^ and *k*_*-*2_ = 6.7 s^−1^ (Fig. 8, *B* and *C*, Table 1). The destabilized GS^I^ state explains why PydC fluorescence is not observed for R129A (Fig. 8*C*). Furthermore, global fitting estimates a rate constant for methylation around 300-fold slower than for WT CcrM, suggesting that the residue R129 interacts with is critical for alignment for catalysis; R129A *k*_3_ = 0.001 s^−1^, WT *k*_3_ = 0.21 s^−1^ (Fig. 8*D*). Again, product release (*k*_4_) was locked at the WT value (1.63 s^−1^). Global fitting of the data for R129A shows that *K*_2_ is now unfavorable because of destabilization of GS^I^ (Fig. 8*E*). Confidence contour analysis for R129A shows that the kinetic parameters are well defined by the data (Fig. S2).

**Figure 9:**
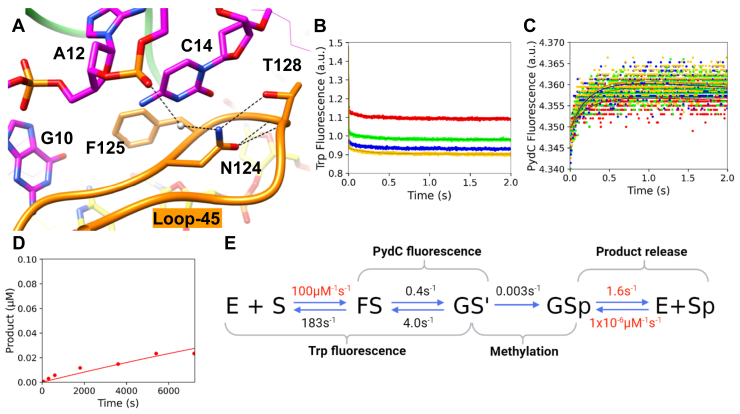
**The N124A mutant destabilizes strand separation, which limits catalysis.***A*, N124 from loop-45 makes a stacking interaction with C_14_ (5′GANTC3′) of the target strand recognition site and makes a water-mediated hydrogen bond to the phosphate backbone of T_13_. N124 also makes interloop hydrogen bonds to T128 of loop-45 and the peptide backbone of loop-45. *B*, N124A can bind DNA, and the protein isomerization event occurs as seen with WT. Trp fluorescence monitored the kinetics of binding and protein isomerization and consisted of N124A (500 nM), C1-DNA (2.5 [*red*], 5.0 [*green*], 7.5 [*blue*], and 10.0 [*yellow*] μM), and SAM (60 μM). *C*, N124A has negligible DNA strand separation, as seen in the low signal to noise of PydC fluorescence. DNA strand–separation kinetics monitored by PydC fluorescence consisted of P1-DNA (1.0 μM), N124A (2.5 [*red*], 5.0 [*green*], 7.5 [*blue*], and 10.0 [*yellow*] μM), and SAM (60 μM). *D*, N124A can methylate DNA but at a slower observed rate than WT. Single-turnover methylation monitored *via* radiochemical methods consisted of C1-DNA (100 nM), N124A (250 nM), and tritiated SAM (15 μM). *E*, global fitting of the data for N124A shows that methylation is reduced relative to WT because of destabilization of the strand-separated state (GS^I^). All experiments (*A*–*C*) were globally fit in KinTek Explorer. Product release was not defined for N124A; therefore, *k*_*4*_ was locked at the WT value (1.63 s^−1^). Rates in *red* were locked during the simulation, whereas rates in *black* were allowed to float. *Solid lines* represent the simulated traces from the global fit.

N124 is a highly conserved residue from loop-45 that does not make any direct hydrogen bonds to nucleic acids (although it makes a water-mediated hydrogen bond with the backbone near A12) but recognizes C_14_ of the recognition site *via* stacking interactions (Fig. 9*A*) and has direct interaction with T128 from the same loop. Equilibrium measurements show that N124A can bind DNA with a similar affinity as WT; N124A *K*_*d*_^app^ = 138 ± 18 nM, WT *K*_*d*_^app^ = 150 ± 5 nM (Fig. S1). Stopped-flow experiments show that N124A displays a reduced *k*_2_ and increased *k*_-2_; N124A *k*_2_ = 0.39 s^−1^ and *k*_-2_ = 3.9 s^−1^, WT *k*_2_ = 23.2 s^−1^ and *k*_-2_ = 6.7 s^−1^ (Fig. 9, *B* and *C*, Table 1). The rate constant for methylation is greatly reduced for N124A with *k*_3_ = 0.003 s^−1^ (Fig. 9*D*, Table 1). Again, product release (*k*_4_) was locked at the WT value (1.63 s^−1^). Global fitting of the data for N124A shows that methylation is reduced relative to WT because of destabilization of the strand-separated state (GS^I^), which perturbs the alignment of catalytic residues required for catalysis (Fig. 9*E*). Confidence contour analysis for N124A shows that the kinetic parameters are well defined by the data (Fig. S2).

**Figure 10:**
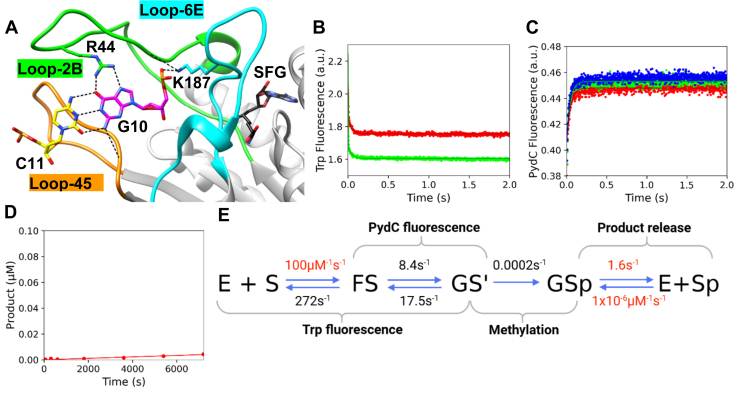
**The R44A mutant destabilizes the strand-separated state (GS^I^), severely disrupting methylation.***A*, R44 from loop-2B makes hydrogen bonds to G_10_ (5′GANTC3′) of the target strand, which is the only base of the recognition site that maintains Watson–Crick base pairing during strand separation. *B*, R44A can bind DNA and undergo the protein isomerization event with similar rates to WT. Trp fluorescence monitored the kinetics of binding and protein isomerization and consisted of R44A (500 nM), C1-DNA (2.5 [*red*] and 5.0 [*green*] μM), and SAM (60 μM). *C*, R44A can strand-separate DNA to a lesser extent than WT, as seen in the diminished amplitude of PydC fluorescence. DNA strand–separation kinetics monitored by PydC fluorescence consisted of P1-DNA (1.0 μM), R44A (5.0 [*red*], 7.5 [*green*], and 10.0 [*blue*] μM), and SAM (60 μM). *D*, R44A cannot methylate DNA, as seen by the lack of product formation. Single-turnover methylation monitored *via* radiochemical methods consisted of C1-DNA (100 nM), R44A (250 nM), and tritiated SAM (15 μM). *E*, global fitting of the data for R44A shows that *K*2 is now unfavorable because of destabilization of GS^I^. All experiments (*A*–*C*) were globally fit in KinTek Explorer. Product release was not defined for R44A; therefore, *k*_4_ was locked at the WT value (1.63 s^−1^). Rates in *red* were locked during the simulation, whereas rates in *black* were allowed to float. *Solid lines* represent the simulated traces from the global fit.

R44 is a highly conserved residue from loop-2B that makes hydrogen bonds to G_10_ of the target strand, which is the only base of the recognition site that maintains Watson–Crick hydrogen bonds during strand separation (Fig. 10*A*). R44A binds DNA with a similar affinity as WT; R44A *K*_*d*_^app^ = 145 ± 9.5 nM, WT *K*_*d*_^app^ = 150 ± 5 nM (Fig. S1). Stopped-flow experiments show that R44A has a destabilized GS^I^ state; R44A *k*_2_ = 8.4s^−1^, *k*_-2_ = 17.5 s^−1^ (Fig. 10, *B* and *C*, Table 1). Furthermore, the rate constant for methylation for R44A is reduced dramatically; R44A *k*_3_ = 0.0002 s^−1^, WT *k*_3_ = 0.21 s^−1^ (Fig. 10*D*). Again, product release (*k*_4_) was locked at the WT value (1.63 s^−1^). Global fitting of the data for R44A shows that *K*_2_ is now unfavorable because of the destabilization of GS^I^ (Fig. 10*E*). Confidence contour analysis for R44A shows that the kinetic parameters are well defined by the data (Fig. S2).

### Loop-2B and loop-45 are conserved across human and animal pathogens

**Figure 11:**
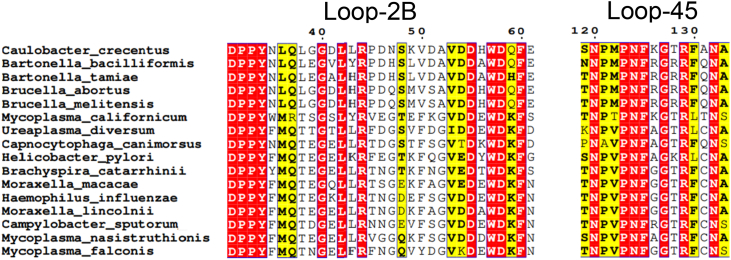
**Loop-2B and loop-45 are highly conserved in CcrM orthologs across pathogenic orthologs.** The C-terminal domain was used as the search seed for this BLAST. Multiple sequence alignment was done using Clustal Omega, and the MSA was visualized using ESPript 3.0 with a similarity color scheme global score of 0.7. Numbering is based on the CcrM sequence from *Caulobacter crescentus*. CcrM, cell cycle–regulated DNA methyltransferase.

**Figure 12:**
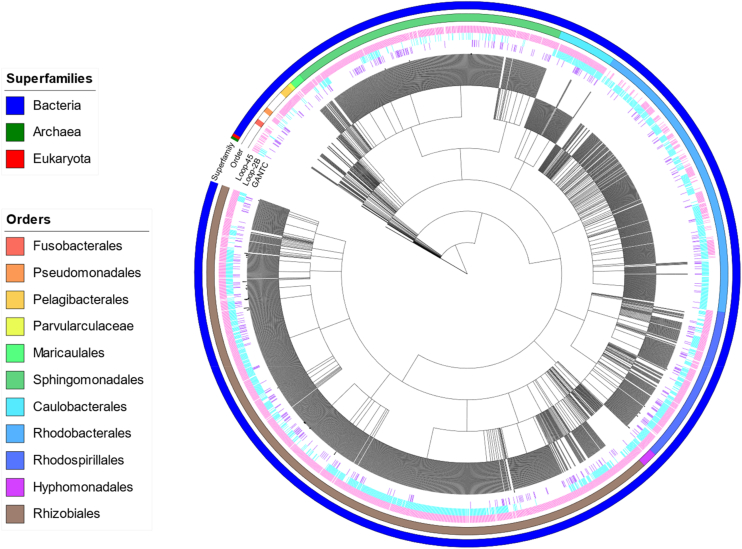
**Presence of loop motif sequences in proteins with an 80 AA consensus sequence.** This phylogenetic tree displays the organisms with proteins that appeared in a ScanProsite search using the 83 AA consensus sequence: [RK]-[VI]-[PAS]-[FML]-x(4)-[EDNS]-x-[GNDH]-x(4)-[GN]-x-x-[LVFI]-x(8,12)-[ACV]-x(4,6)-[DNGS]-[GAS]-x(9,16)-S-I-H-x(12,14)-N-G-[WF]-x(14,16)-[DN]-x-x-R. The superfamilies of the organisms are indicated in the outermost ring of annotations. The orders containing more than five organisms are indicated by the second ring. The presence of loop-45 is indicated by a *pink line* in the third ring, and the presence of loop-2B is indicated by a *cyan line* in the fourth ring, and confirmed GANTC recognizers (REBASE) are indicated by a *violet line* in the innermost ring of annotations. The tree was created using the National Center for Biotechnology Information Common Tree and visualized with iTOL. All nodes are collapsed to the species level. All the methyltransferases with known recognition sequences in this tree recognize GANTC. This annotation was provided by REBASE. The unlabeled methyltransferases have unidentified recognition sequences and are not annotated in REBASE. There are no other recognition sequences that we have identified other than GANTC.

**Figure 13:**
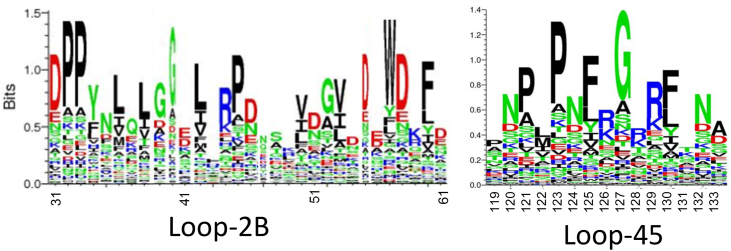
**Motif organization and sequence logos of loop-2B and loop-45.** The logos were constructed from the methyltransferases in the phylogenetic tree. Residue labeling is based off the CcrM sequence. The sequences were aligned with Clustal Omega, and the logos were constructed with Seq2Logo. CcrM, cell cycle–regulated DNA methyltransferase.

We sought to further understand the importance of these structural elements that regulate strand separation (loop-2B and loop-45) by looking at these motifs in CcrM orthologs. These residues and loops are conserved across DNA methyltransferases in human and animal pathogens, including *Brucella melitensis*, *Mycoplasma falconis*, *Mycoplasma nasistruthionis*, *Campylobacter sputorum*, *Moraxella lincolnii*, *Haemophilus influenzae*, *Moraxella macacae*, *Brachyspira catarrhinii*, *Helicobacter pylori*, *Capnocytophaga canimorsus*, *Ureaplasma diversum*, *Mycoplasma californicum*, *B. melitensis*, *Brucella abortus*, *Bartonella tamiae*, *Bartonella bacilliformis*, and others (Fig. 11). All identified orthologs have the CcrM 83 amino acid C-terminal domain, and multiple sequence alignment (MSA) reveals the high conservation of loop-2B and loop-45 (Fig. 11). Organisms that contain a CcrM ortholog with a C-terminal domain, loop-2B, and loop-45 are found across many orders of bacteria, as well as some eukaryotes and some archaea (Fig. 12). The variation of these loops is shown *via* a SeqLogo (Fig. 13).

### Orphan orthologs are more discriminating than orthologs from restriction–modification systems

**Figure 14:**
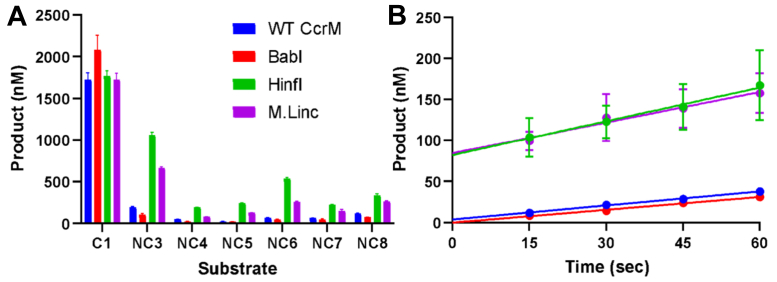
**CcrM orphan orthologs are more discriminating than orthologs from RM systems, and orthologs from RM systems display a steady-state burst.** CcrM and BabI are orphan MTases, whereas HinfI and M.Linc are MTases within restriction–modification (RM) systems. *A*, substrate specificity for CcrM orthologs. CcrM and BabI are orphan DNA methyltransferases and are highly discriminating against noncognate substrates. Each reaction consisted of CcrM (2.5 μM), DNA substrate (1.0 μM), tritiated SAM (60.0 μM), and one 120-min time point was spotted in triplicate. *B*, steady-state burst experiment with CcrM orthologs. CcrM and BabI do not display a steady-state burst, indicating that a step preceding product release is rate limiting. M.Linc and HinfI are MTases from RM systems, and they display the typical steady-state burst. A burst amplitude of approximately 100 is indicative of 100 nM of dimeric enzyme. A steady-state burst indicates that there is rapid enzyme-bound product formation, and product release is rate limiting. This relationship suggests that discrimination is slower than product release for orphan enzymes, whereas product release is rate limiting for enzymes from RM systems. Each burst reaction consisted of enzyme (200 nm), C1-DNA (3 μM), and tritiated SAM (15 μM). CcrM, cell cycle–regulated DNA methyltransferase.

We also sought to understand how orthologs within different biological contexts (orphans or part of restriction–modification [RM] systems) differed mechanistically. We carried out specificity analysis with a CcrM orphan ortholog and related bacterial RM enzymes (Fig. 14*A*). Noncognate DNA substrates (NC3, NC4, NC5, NC6, NC7, and NC8) contain one base substitution in the recognition site, and C1 is the cognate substrate (Table S1). BabI is an orphan ortholog, whereas HinfI and *M. lincolnii* (M.Linc) are orthologs within RM systems. CcrM and BabI are more discriminating against noncognate substrates than HinfI and M.Linc (Fig. 14*A*). CcrM and BabI do not display a steady-state burst, indicating that a step preceding product release is rate limiting (Fig. 14*B*), unlike M.Linc and HinfI, which show the typical burst. This suggests that enzymes from RM systems may have less stringent requirements for selectivity than orphan enzymes that are responsible for gene expression and cell-cycle regulation.

## Discussion

The DNA strand–separation mechanism used by CcrM is thought to be essential for its high sequence discrimination (33, 34). Here, we sought to better understand this process through structural, kinetic, and evolutionary analysis. This has relevance both for our understanding of a new DNA recognition mechanism and how the kinetic partitioning of reaction intermediates can contribute to enzyme specificity. A mechanistic understanding of how enzymes induce conformational changes in their substrates is particularly important, however challenging, since the obligate intermediates are transient and require sophisticated probes and global data fitting methods to allow kinetic assignments. Moreover, there are now numerous examples showing that such transitions can be key determinants of enzyme specificity, and rigorous global fitting has been essential to resolving these pathways (35, 36, 37, 38). Understanding such mechanisms can lead to the design of highly specific cell biology tools and therapeutics (39).

We utilized stopped-flow fluorescence to quantify the rates of protein conformation changes and DNA strand separation as well as radiochemical methods to monitor methyl transfer. Global fitting of all the data resolved the rate constants that were assigned to transitions between known intermediates. Specifically, we sought to understand the roles of loop-2B and loop-45 in regulating DNA strand separation through an analysis of F125L, F125W, F125A, R129A, N124A, and R44A CcrM mutants.

Loop-2B and loop-45 are positioned within the strand-separated DNA, make interactions with target-strand bases, and appear to be stabilized by interactions with other protein residues (Figs. 1 and 2, 32). The structural positioning of these loops suggests that they are important motifs for supporting DNA strand separation, and we showed previously that strand separation is tightly coupled with substrate discrimination (34). We selected residues for mutational analysis by their high degree of conservation and structural implication in strand separation. R44, N124, and R129 make specific interactions to target strand bases in the strand-separated (GS^I^) state (Figure 8, Figure 9, Figure 10). We previously interrogated CcrM’s DNA specificity by making noncognate nucleic acid substitutions in the recognition site (34). Here, our focus is on protein residues and motifs that could either contribute to recognition (R44A, N124A, and R129A) or play a role in generating or stabilizing the strand separation (F125) without making base-specific interactions.

**Figure 15:**
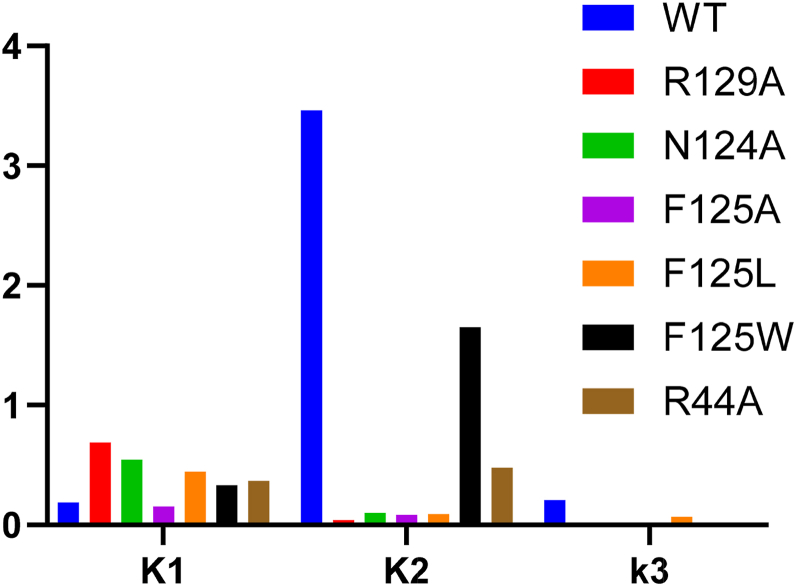
**Comparison of equilibrium constants between WT and loop mutants.** All mutants have similar *K*_1_ relative to WT, indicating that loops do not contribute significantly to stabilizing initial binding of the enzyme to DNA. All mutants, except for F125W, have a reduced *K*_2_, indicating a destabilized GS^I^ intermediate. All mutants have decreased methylation (*k*_3_), which is not an equilibrium constant but was included for reference.

Mutational analysis of the highly conserved F125 revealed interesting functional responsibilities of this residue. F125L destabilizes the GS^I^ intermediate (Fig. 5) and decreases the rate of catalysis (Fig. 5). Noteworthy, the equilibrium constant *K*_2_ favors the reverse direction in the pathway, compared with WT, which favors going forward (F125L *K*_2_ = 0.09, WT *K*_2_ = 3.5, Fig. 15, Table S3). Thus, global fitting suggests that this residue contributes to both generation and stabilization of DNA strand separation. Consistent with this dual role, the F125A mutant reduced hydrophobicity further, and the resultant equilibrium constant *K*_2_ (0.09) also favors the reverse direction (Fig. 15, Table S3).

We further interrogated the role of residue 125 in loop-45 by introducing a tryptophan residue at this position with the F125W mutant. The F125W kinetic model reveals that the formation and stabilization of the GS^I^ species is slightly lessened with this mutant (*K*_2_ = 1.7) than WT CcrM (*K*_2_ = 3.5) (Table 1) (Fig. 15, Table S3). However, the F125W GS^I^ intermediate has a 10-fold reduced rate of methylation (*k*_3_) (Table 1, Table S3), suggesting a steric effect that interferes with alignment of substrates and catalytic residues. These data support a model where loop-45 contributes to generating partial strand separation and that F125 is essential for separating the strands to the correct position so that other residues can make base-specific interactions that are essential for methylation.

In summary, global data fitting resolves that *K*_2_ is the equilibrium constant most perturbed by mutations at F125, with hydrophobic residues aiding in generation and stabilization of DNA strand separation. Furthermore, F125 does not appear to make any base-specific interactions, which can contribute to recognition by stabilizing the GS^I^ intermediate that precedes methylation in the CcrM kinetic model.

On the other hand, we also wanted to understand how conserved loop residues that do make base-specific interactions contribute to strand separation *via* recognition interactions. We approached this by designing loop mutants that make base-specific contacts to target strand bases within the recognition site. R129 in loop-45 makes two hydrogen bonds to T_13_ within the target strand recognition site (Fig. 8*A*). PydC fluorescence changes, which track DNA strand separation, are not observed for R129A because of the inability to form and stabilize the strand-separated intermediate (GS^I^). The result of the destabilized GS^I^ is minimal product formation for R129A (Fig. 8*D*). R129A is unable to recognize T_13_, thus perturbing the protein’s ability to form and stabilize the strand-separated state (GS^I^) (Fig. 8*E*). R129A indicates that recognition interactions from loop-45 contribute to the formation of the strand-separated state in addition to stabilization.

N124 is another highly conserved residue from loop-45 that does not make bonds to any specific nucleobases but contributes to recognition of C_14_ (5’GANTC’3) *via* stacking interactions (Fig. 9*A*). N124A results in the destabilization of the intermediate GS^I^ and disfavors going forward in the pathway, as seen in the mutant kinetic model (Fig. 9). The destabilization of GS^I^ by N124A supports our hypothesis that recognition interactions are essential to generate and stabilize strand separation, which is essential for catalysis.

Interestingly, R44A shows a small PydC signal indicating that this mutant is capable of strand separation (Fig. 10*C*). R44 stands out amongst the residues selected for mutation because it recognizes G_10_ of the 5’GANTC’3 recognition site. G_10_ is the only base within the recognition site where Watson–Crick base pairing is maintained in the strand-separated GS^I^ state. R44A has similar strand-separation forward kinetics as WT (R44A *k*_2_ = 8.4 s^−1^, WT *k*_2_ = 23.2 s^−1^), but the GS^I^ state is destabilized as seen in the reverse kinetics (R44A *k*_-2_ = 17.5 s^−1^, WT *k*_-2_ = 6.7 s^−1^) (Table 1). R44’s recognition of G_10_ contributes to the generation and stabilization of GS^I^, and DNA strand separation can begin prior to R44’s recognition of G_10_. In agreement with the other loop mutants that disable recognition, R44A’s *K*_2_ (0.48) is sevenfold reduced from WT (3.5) (Fig. 15, Table S3). Analysis of R44A supports our model in which recognition interactions are essential for generation and stabilization of the strand-separated state, which is essential for catalysis.

Importantly, we attempted to incorporate the anisotropy binding data (Fig. S1) in our WT and mutant global fitting models; however, a good fit was not resolved. One explanation for this fitting inconsistency is that the anisotropy experiment used SAH as a nonreactive cofactor, whereas the Trp kinetic experiment used SAM as a cofactor that allows the reaction to go to completion. We showed previously that a cofactor affects the kinetics of DNA strand separation (34). A second explanation is that the anisotropy experiment used 10 nM FAM-labeled DNA with 10 nM^−1^ μM CcrM, whereas the Trp kinetic experiment used 500 nM CcrM and 2.5 to 10 μM unlabeled DNA.

Together, the results with N124A, R129A, and R44A, which perturb base-specific recognition between loop residues to target strand bases, reveal that these interactions contribute to formation and stabilization of the strand-separated intermediate (GS^I^). This further supports our previous understanding of this system, where we showed that DNA strand separation is tightly coupled with substrate discrimination (34). In our previous work, we approached this relationship from the perspective of the DNA by building a model with WT CcrM and noncognate DNA (34). Here, we approached this from the perspective of the protein, where we built models with CcrM mutants and cognate DNA. In both cases, the substrate recognition at a single base is removed, and the resulting steps in the enzyme kinetic model are the strand-separation steps in the forward and reverse directions. The perturbations of DNA strand separation are also correlated with methylation efficiency (Table 2), suggesting that precise positioning of the strand-separated intermediate is crucial for proper alignment of catalytic residues.

We previously showed that the C-terminal domain of CcrM is essential for binding and DNA strand separation (33). The C-terminal domain is dispersed across CcrM orthologs in several orders of bacteria, some eukaryotes, and some archaea (33). We wanted to see how these loops were included or varied in these orthologs that also contained the unique C-terminal domain. Organisms that contain a CcrM ortholog with a C-terminal domain, loop-2B, and loop-45 are found across many orders of bacteria as well as some eukaryotes and some archaea (Figs. 11 and 12). Together, the C-terminal domain, loop-2B, and loop-45 are essential for generating strand separation and stabilizing the strand-separated intermediate and thus contribute to regulating CcrM’s extreme substrate fidelity. This unique substrate discrimination mechanism is complex and useful for understanding how enzymes have evolved unique mechanisms for regulating cellular processes.

CcrM and the orphan ortholog (BabI) stand out mechanistically from orthologs from RM systems (HinfI and M.Linc). CcrM and BabI’s higher level of substrate discrimination and lack of a steady-state burst (Fig. 14) indicate that it may be important for specificity to have a limiting step at or before the chemical step, which for these systems is largely irreversible. HinfI and M.Linc’s pre–steady-state burst suggests that these enzymes might have evolved to favor faster catalysis to trap the methylated product, rather than higher levels of discrimination, which could lower their efficiency. In addition, enzymes from RM systems may have less stringent requirements for selectivity resulting in host protection than orphan enzymes that are responsible for gene expression and cell-cycle regulation.

In summary, the motifs and mechanisms that govern DNA strand separation and substrate fidelity are relevant to other proteins, such as CRISPR–Cas9 and RNA polymerase sigma factor (39, 40). In contrast to CRISPR and sigma factor, CcrM’s DNA strand separation is tightly coupled to the extreme sequence selectivity, providing a compelling opportunity to study this newly described recognition strategy (39). The results presented here for CcrM can be useful for understanding substrate discrimination in other systems, such as CRISPR–Cas9, where gene editing tools require overcoming barriers of selectivity between enzymes and DNA substrates.

## Experimental procedures

### DNA

DNA substrates were obtained from Integrated DNA Technologies and the Yale Keck Oligo Synthesis Facilities. The oligos were annealed at 95 °C for 5 min in 10 mM Tris–HCl, 50 mM NaCl, 1 mM EDTA, pH 8, and subsequently cooled passively to room temperature. Annealing was analyzed by 10% native PAGE. Gels were imaged on a Bio-Rad Gel Doc EZ Imager. Densitometry was performed with FIJI ImageJ, which determined a >98% annealing success. Substrate DNA sequences can be found in Table S1.

### Site-directed mutagenesis

Mutant plasmids were constructed using the Agilent Quikchange Lightning Site-Directed Mutagenesis Kit. The primer sequences used in the PCRs can be found in Table S2. Mutant plasmids were transformed into XL10 Ultracompetent *Escherichia coli* cells (Agilent), and the plasmid was isolated using an Agilent Mini Prep Kit. Plasmids were sequenced by Laragen, Inc, and confirmed plasmids were transformed into the New England Biolabs Nico21 (DE3) *E. coli* cells for protein production.

### Protein purification

WT CcrM and mutant plasmids containing kanamycin resistance were cloned as described previously (33). Protein production and purification were also previously reported (33). Specifically, plasmids were transformed into NiCo21(DE3) cells (New England Biolabs) and grown overnight at 37 °C on LB-agar plates with 30 μg/ml kanamycin. A single colony was collected and grown for 16 h in LB medium at 37 °C on a New Brunswick G10 Gyrotory shaker at 230 rpm. One-liter cultures containing 30 μg/ml kanamycin were then inoculated with the 16 h preculture and grown at 37 °C at 230 rpm until an absorbance of 0.9 to 1.0 at 600 nm. The cultures were placed on ice for 10 min to facilitate their reaching of room temperature, followed by CcrM expression induced with 1.68 mM IPTG at 225 rpm and 25 °C for 3 h. Cells were pelleted at 4 °C, 5000 rpm for 15 min in a J2-21 centrifuge (Beckman Coulter) with a JA-10 rotor and stored at −80 °C. Cells were resuspended in 50 ml of lysis buffer (50 mM Hepes, 400 mM NaCl, 10% glycerol, 10 mM imidazole, pH 8.0). After the addition of PMSF to a concentration of 1 mM, the solution, maintained at below 4 °C in a water–ice slurry, was sonicated with a Branson digital sonifier at 70% amplitude for 1.5 min in 2 s increments. The lysate was cleared by centrifugation at 4 °C with a JA-20 rotor at 11,000 rpm for 1 h, and the supernatant was purified at 1 ml/min on a GE 1 ml HisTrap column using an ÄKTA Start FPLC system. The bound protein was washed with 9.5 column volumes of lysis buffer and eluted with a linear gradient from 10 mM to 250 mM imidazole over nine column volumes collected in 30 1 ml fractions. Pure CcrM fractions were identified by SDS-PAGE, then pooled, concentrated, and buffer exchanged into 50 mM Hepes, 400 mM NaCl, 1 mM DTT, 0.5 mM EDTA, 10% glycerol, pH 8.0, using 10 kDa cutoff Amicon Ultra 15 ml centrifugal filters. The pure protein was then stored at −80 °C in 25 mM Hepes, 200 mM NaCl, 0.5 mM DTT, 0.25 mM EDTA, 25% glycerol, pH 8.0. Enzyme concentrations reported throughout this work refer to the monomer concentration.

### Kinetic measurements by Trp fluorescence

Kinetic constants were measured on an Applied Photophysics SX.18MV stopped-flow spectrometer, temperature controlled to 22 ± 1 °C and using 296 nm excitation and a 320 nm emission cutoff filter. Final concentrations after 1:1 mixing were 500 nM enzyme, 60.0 μM SAM, and DNA that varied at 2.5, 5.0, 7.5, and 10 μM. Experiments were carried out in reaction buffer: 100 mM Hepes, 1 mM EDTA, 20 mM NaCl, 2 mM DTT, pH 8. Kinetic traces were collected in triplicate and averaged. Time-dependent data were corrected for the measured dead time of the stopped-flow instrument (2.5 ms) by adding 2.5 ms to the time scale to shift the data and reflect the absence of data in the first 2.5 ms. For initial characterization before global fitting, data were fit to double-exponential functions in KinTek Global Kinetic Explorer. The function used for fitting was y = a_0_ + a_1_(1 – e^−b1t^) + a_2_(1 – e^−b2t^), where y = fluorescence intensity (arbitrary units), t = time (seconds), a_1_ = the amplitude of the first phase, b_1_ = the rate of the first phase, a_2_ = the amplitude of the second phase, b_2_ = the rate of the second phase, and a_0_ = the initial fluorescence amplitude (arbitrary units). The initial fluorescence amplitudes for kinetic fluorescence data are arbitrary.

### Kinetic measurements by PydC fluorescence to track DNA strand separation

PydC fluorescence kinetics were measured in reaction buffer at 22 ± 1 °C using an Applied Photophysics SX.18MV stopped-flow spectrometer. The excitation wavelength was 350 nm, and emission was collected with a 385-nm cutoff filter. The concentrations after 1:1 mixing were 2.5, 5.0, 7.5, and 10 μM CcrM, 60.0 μM SAM, and 1 μM dsDNA. Data collection and analysis were identical to the procedure used for the Trp fluorescence experiments.

### Radiochemical methylation assay

Single-turnover methylation reactions consisted of 250 nM enzyme, 100 nM DNA, and 15 μM SAM, using hemimethylated double-stranded substrates. Tritiated SAM in all instances had a specific activity of 82.7 mCi/mmol. Reactions were initiated with enzymes. Five microliters of the reaction sample were spotted in triplicate onto GE Amersham Hybond-XL nylon membranes, followed by three 5 min washes in wash buffer (50 mM KH_2_PO_4_). The washes were followed by a 5 min dehydration step with 80% EtOH, another wash for 5 min in 100% EtOH, and a final drying step for 10 min under a heat lamp. Samples were then placed into scintillation vials containing 3 ml of BioSafe II scintillation cocktail. Radiochemical data were generated with a Hidex 300 SL scintillation counter. Data for the single-turnover reaction were background-subtracted and fit to a one-phase decay model in GraphPad Prism 10.0.2.

### Global fitting

Global data fitting was performed in KinTek Global Kinetic Explorer, version 11.0.1. The reaction scheme used as the unifying model to describe the experimental data was E + S ⇌ FS ⇌ GS^I^ ⇌ GSp ⇌ E + Sp. Data from three experiments were input and modeled to reproduce how the data were collected. Time-dependent data were corrected for the measured dead time of the stopped-flow instrument (2.5 ms) by adding 2.5 ms to the time scale to shift the data and reflect the absence of data in the first 2.5 ms. Each experiment had a unique observable signal, which relates the experimental data to the model. For example, experiment 1 is PydC kinetics over 2 s, where PydC fluorescence depends on the following observable signal: a1∗ (S + FS + e ∗ GS^I^ + f ∗ GSp + h ∗ Sp). This observable signal describes that GS^I^ partially contributes to the change in the PydC signal by a factor, *e*, whereas GSp also contributes to the signal, scaled by a factor *f*. Experiment 2 is the radiochemical methylation assay where the observable signal contains all product states plus a small background, GSp + Sp + bkg2. Experiment 3 is Trp kinetics, which depend on the following observable signal: a3 ∗ (E + k ∗ FS + m ∗ GS^I^ + n ∗ GSp). Data from all four experiments were fit globally based on numerical integration of the rate equations (computer simulation). Initial values were estimated based on the fitting of each experiment to exponential equations. Fluorescence scaling factors were applied to the data in experiments 1 and 3 to normalize small variability in the starting amplitudes for each trace within a concentration series. In some cases, scaling factors were close to unity and therefore did not define the concentration and time dependence of these species. Some values were locked during the simulation, whereas others were allowed to float, as described in the text. Locked values were chosen based on the parameter’s lower limit, above which there was no effect on the fitted curves. Individual models were built for F125A, F125L, F125W, R129A, R44A, and N124A with identical model architecture for direct comparison to the WT model. Confidence contour analysis was performed with the 1D FitSpace function in KinTek Explorer and outlines the space over which parameters can vary. Values reported in Table 1 represent the best fit and 95% confidence interval for each parameter allowed to float in the fitting.

$$\text{Equations}\;\text{used}\;\text{to}\;\text{derive}\;k_{\text{cat}},K_{M},\text{and}\;k_{\text{cat}}/K_{M}$$

$$k_{\text{cat}}=\left[k_{2}k_{3}k_{4}\right]/\;\left[\left(k_{2}\left(k_{3}+k_{-3}+k_{4}\right)+k_{-2}\left(k_{-3}+k_{-4}\right)+k_{3}k_{4}\right)\right]$$

$$K_{M}=\left[k_{-1}\left(k_{-2}\;k_{-3}+k_{-2}\;k4+k_{3}k_{4}\;\right)+k_{2}k_{3}k_{4}\right]/\;\left[k_{1}\left(\left(k_{2}\left(k_{3}+k_{-3}+k_{4}\right)k_{-2}\left(k_{-3}+k_{4}\;\right)+k_{3}k_{4}\right)\right)\right]$$

$$k_{\text{cat}}/K_{M}=\left[k_{1}k_{2}k_{3}k_{4}\right]/\;\left[\left(k_{-1}\left(k_{-2}k_{-3}+k_{-2}k_{4}+k_{3}k_{4}\right)+k_{2}k_{3}k_{4}\right)\right]$$

**Table 1:** Summary of rate constants derived from global fitting for loop mutants

Enzyme	1/*K*_*1*_ (μM)	*k*_2_ (s^−1^)	*k*_-2_ (s^−1^)	*k*_3_ (s^−1^)
WT	5.28 (5.20–5.37)	23.2 (22.9–23.6)	6.7 (6.55–6.82)	0.21 (0.20–0.22)
F125L	2.24 (2.23–2.24)	1.57 (1.50–1.64)	17.3 (16.4–18.1)	0.069 (0.062–0.078)
F125A	6.42 (6.38–6.48)	0.57 (0.48–0.74)	6.6 (5.9–8.0)	0.0025 (0.0021–0.0029)
F125W	3.00 (2.90–3.00)	17.5 (17.1–17.3)	10.6 (10.5–10.6)	0.018 (0.015–0.018)
R129A	1.45 (1.42–1.49)	0.20 (0.17–0.24)	5.0 (4.4–6.0)	0.0009 (0.00087–0.001)
N124A	1.83 (1.81–1.84)	0.39 (0.34–0.42)	3.9 (3.47–4.23)	0.003 (0.00275–0.00301)
R44A	2.72 (2.70–2.74)	8.4 (7.6–9.4)	17.5 (15.9–19.7)	0.000153 (0.00015–0.00016)

Rate constants were derived from global fitting, and lower and limits were derived from confidence contour analysis (Fig. S2). *k*_1_ was locked at 100 μM^−1^ s^−1^ in the fitting (rapid equilibrium), and *k*_*-*1_ was allowed to float, determining *K*_1_. The chemical step (*k*_3_) was assumed to be irreversible (*k*_*-*3_ = 0). Product release (*k*_4_) was locked in the fitting at the value determined for WT CcrM (1.63 s^−1^) as it was not defined for any of the mutants. Product binding (*k*_*-*4_) was locked at 1 × 10^−6^ μM^−1^ s^−1^ as a minimal value.

### Structural images

Structural images were made with UCSF Chimera (Protein Data Bank ID: 6PBD (31)).

### BLAST

The 83-amino acid C-terminal domain was used as the search seed for the BLAST using the UniProt database, BLOSUM62 matrix, and E-threshold = 10. The organisms we identified as human and/or animal pathogens were included in the MSA. MSA was performed with Clustal Omega, and the MSA was visualized using ESPript 3.0 with a similarity color scheme global score of 0.7. Numbering is based on the CcrM sequence from *Caulobacter crescentus*.

### Phylogenetic tree

The phylogenetic tree displays the organisms with proteins that appeared in a ScanProsite search using the 83-amino acid consensus sequence: [RK]-[VI]-[PAS]-[FML]-x(4)-[EDNS]-x-[GNDH]-x(4)-[GN]-x-x-[LVFI]-x(8,12)-[ACV]-x(4,6)-[DNGS]-[GAS]-x(9,16)-S-I-H-x(12,14)-N-G-[WF]-x(14,16)-[DN]-x-x-R. The superfamilies of the organisms are indicated in the outermost ring of annotations. The orders containing more than five organisms are indicated by the second ring. The presence of loop-45 is indicated by a pink line in the third ring, and the presence of loop-2B is indicated by a cyan line in the fourth ring, and confirmed GANTC recognizers (REBASE) are indicated by a violet line in the innermost ring of annotations. The tree was created using the National Center for Biotechnology Information Common Tree and visualized with iTOL. All nodes are collapsed to the species level. All the methyltransferases with known recognition sequences in this tree recognize GANTC. This annotation was provided by REBASE. The unlabeled methyltransferases have unidentified recognition sequences and are not annotated in REBASE. There are no other recognition sequences that we have identified other than GANTC.

## Data availability

All data will be made available by emailing the corresponding author: reich@chem.ucsb.edu.

## Supporting information

This article contains supporting information.

## Conflict of interest

K. A. J. is the President of KinTek Corporation, which provided the KinTek Explorer software used in this study.
