# Supplementary material for: Protein loops are major contributors to DNA strand separation and high-fidelity substrate recognition for DNA methyltransferase CcrM
Source: J Biol Chem. 2026 Mar 24;302(5):111398. doi: 10.1016/j.jbc.2026.111398 (PMC13092597; doi:10.1016/j.jbc.2026.111398)
Supplement: Supplementary Figures and Tables [file mmc1.docx]

**Supplementary Information**


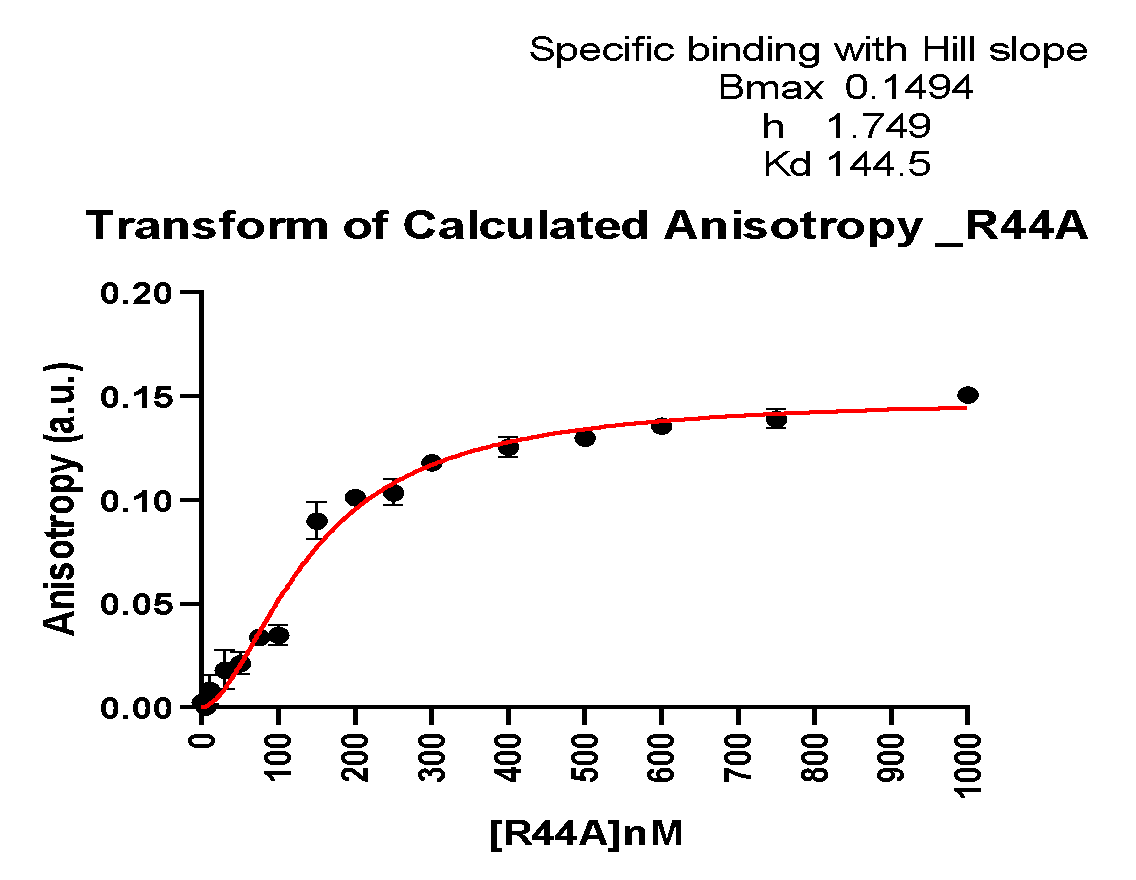

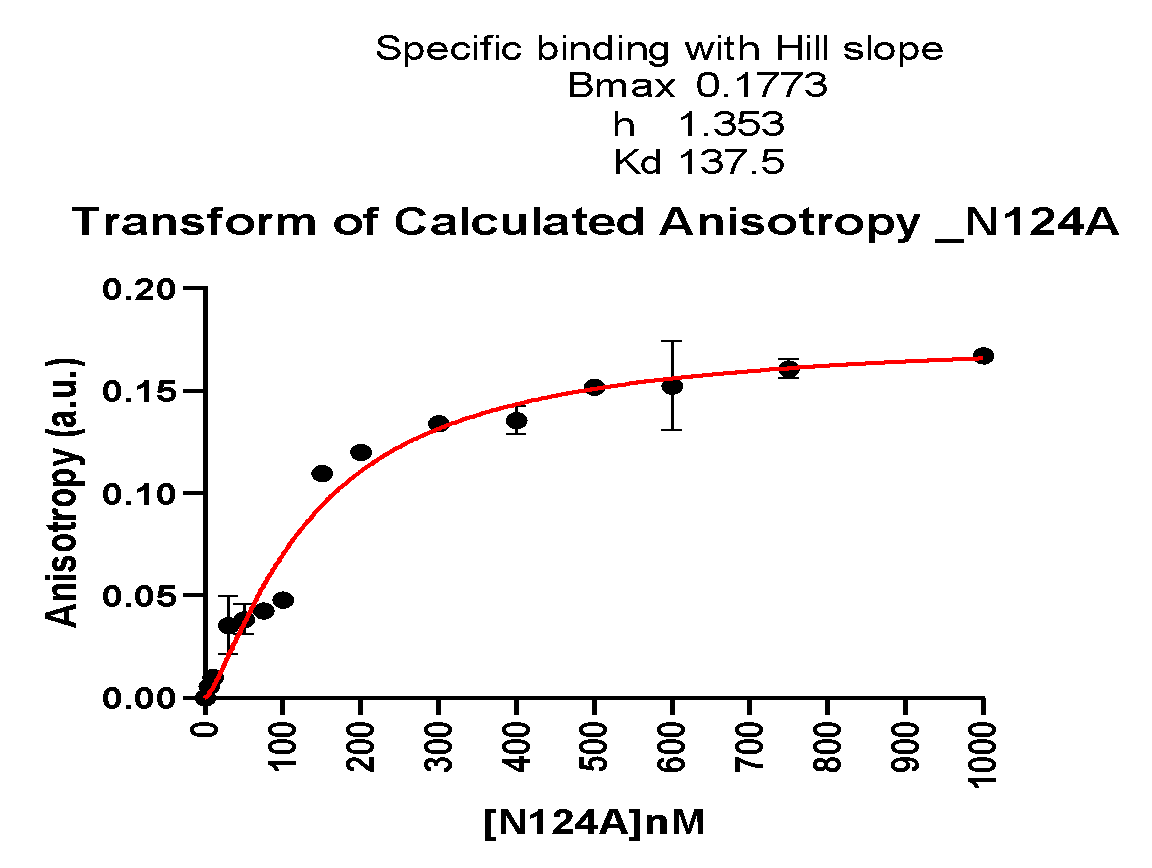

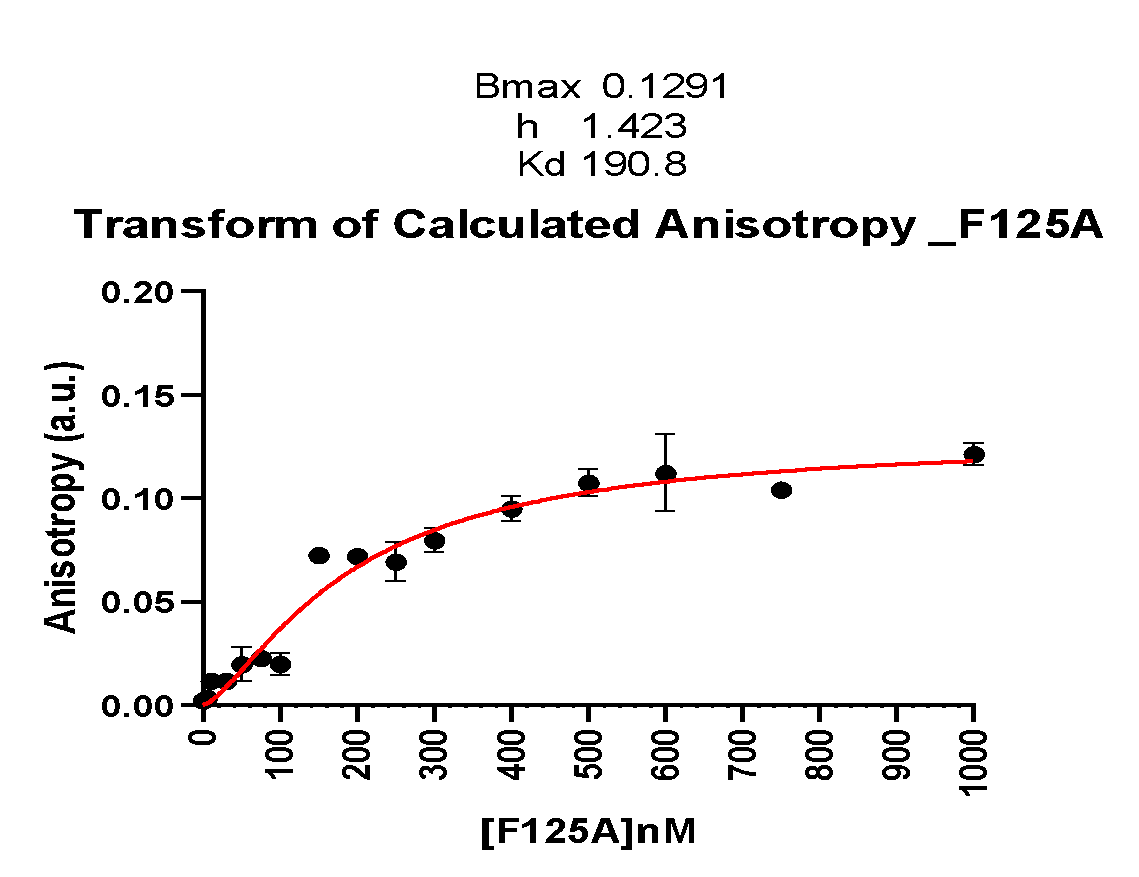

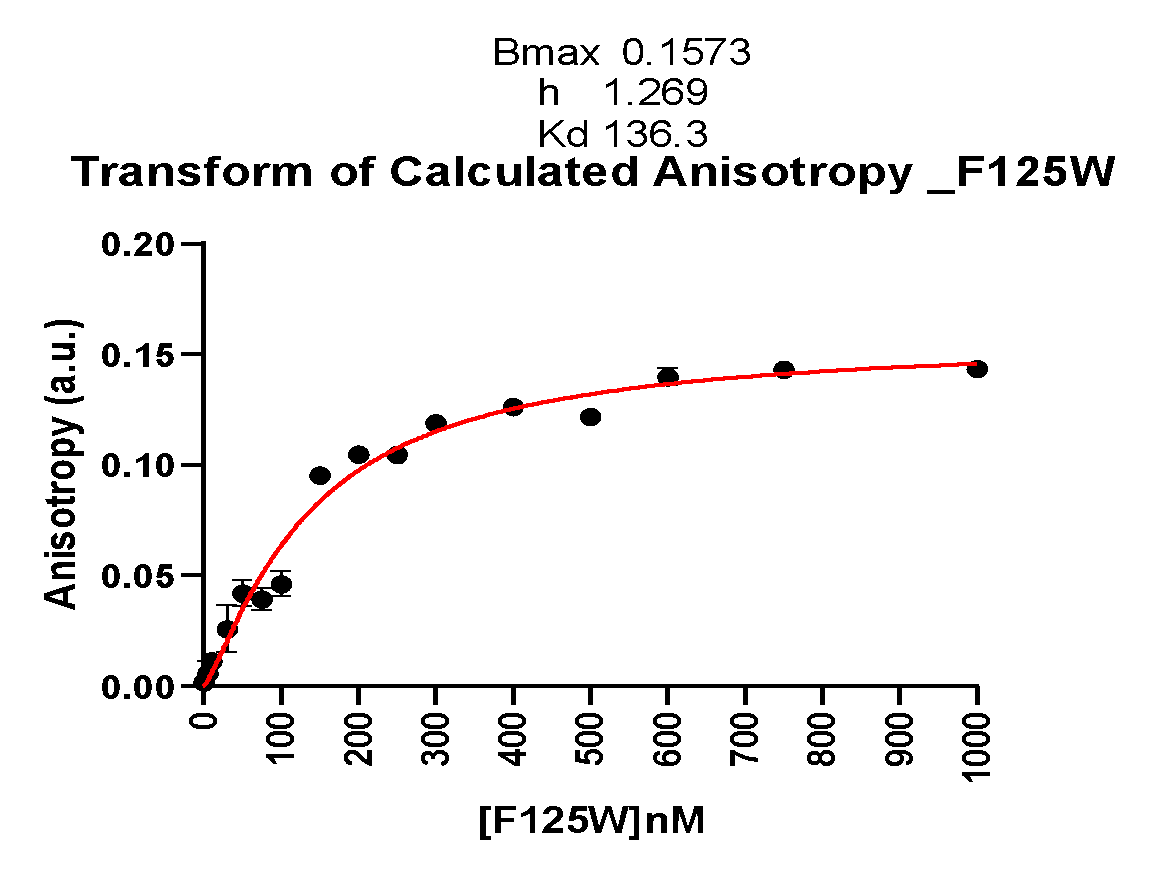

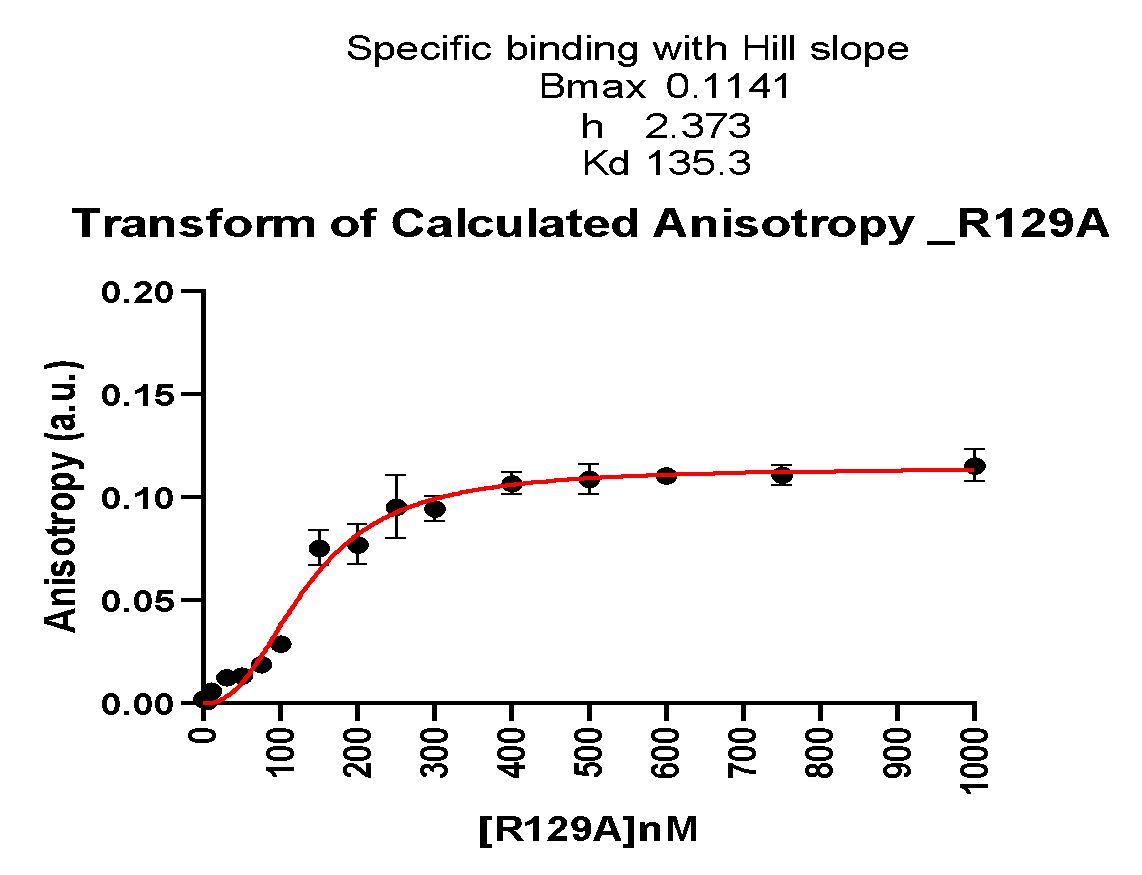

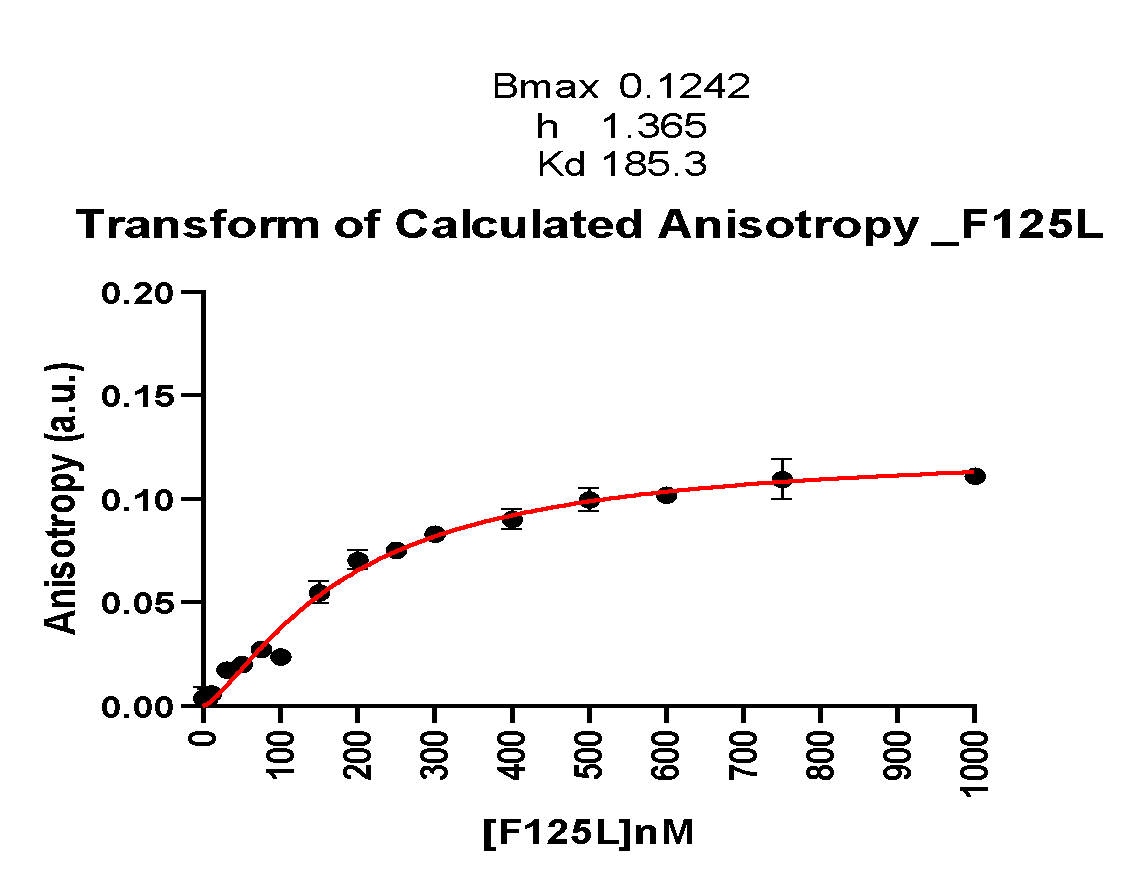


**A**

**B**

**D**

**E**

**F**

**C**

**SI Figure 1. Fluorescence anisotropy to determine the K_d_^app^ of loop mutants for DNA.** Anisotropy conditions were 10 nM FAM-tagged DNA, 15 µM SAH, and varying CcrM monomer concentrations (1, 10, 30, 50, 75, 100, 150, 200, 250, 300, 400, 500, 600, 750, 1000 nM). Importantly, catalysis was prevented by using SAH instead of SAM. **A.** R44A K_d_^app^ = 144.5 ± 9.5, h=1.8. **B.** N124A K_d_^app^ = 137.5 ± 17.8, h = 1.4. **C.** R129A K_d_^app^ = 135.3 ±7.1, h = 2.4. **D.** F125A K_d_^app^ = 190.8 ±30.4, h = 1.4. **E.** F125L K_d_^app^ = 185.3 ±19.1, h = 1.4. **F.** F125W K_d_^app^ = 136.3 ±15.7, h = 1.3. Data were fit in Graphpad Prism 10.0.2 to a specific binding with Hill slope model where Anisotropy = Anisotropy_max_*[CcrM]^h^ /(K_d_^h^ + [CcrM]^h^.

| **DNA name and sequence** | C1 5’-TCACTGTACTCTGACTCGCCTGACATGAC-3’  3’-AGTGACATGAGACTG**M**GCGGACTGTACTG-5’  P1 5’-TCACTGTACTCTGA**P**TCGCCTGACATGAC-3’  3’-AGTGACATGAGACTG**M**GCGGACTGTACTG-5’  NC3 5’-TCACTGTACTCTCACTCGCCTGACATGAC-3’  3’-AGTGACATGAGAGTG**M**GCGGACTGTACTG-5’  NC4 5’-TCACTGTACTCTGACTAGCCTGACATGAC-3’  3’-AGTGACATGAGACTG**M**TCGGACTGTACTG-5’  NC5 5’-TCACTGTACTCTGACTGGCCTGACATGAC-3’  3’-AGTGACATGAGACTG**M**CCGGACTGTACTG-5’  NC6 5’-TCACTGTACTCTGACTTGCCTGACATGAC-3’  3’-AGTGACATGAGACTG**M**ACGGACTGTACTG-5’  NC7 5’-TCACTGTACTCTAACTCGCCTGACATGAC-3’  3’-AGTGACATGAGATTG**M**GCGGACTGTACTG-5’  NC8 5’-TCACTGTACTCTTACTCGCCTGACATGAC-3’  3’-AGTGACATGAGAATG**M**GCGGACTGTACTG-5’ |
| --- | --- |

**SI Table 1. Names and sequences of 29mer DNA substrates.** C1 is cognate DNA, P1 is cognate DNA with PydC inserted at the N-position of the recognition site, NC3, NC4, NC5, NC6, NC7, and NC8 are different noncognate DNA sequences. P = Pyrrolo-dC, M = N6-methyl adenine, red bases = noncognate substitutions.

| Primer | Sequence 5’->3’ |
| --- | --- |
| R44A _ Forward | ggaattgtcgggagccaggaggtccccg |
| R44A _ Reverse | cggggacctcctggctcccgacaattcc |
| R129A _ Forward | ggcgttggcgaaggcggtgcccttgaag |
| R129A _ Reverse | cttcaagggcaccgccttcgccaacgcc |
| N124A _ Forward | gggtgcccttgaaggcgggcatcgggttgg |
| N124A _ Reverse | ccaacccgatgcccgccttcaagggcaccc |
| F125A _ Forward | cccgatgcccaacgccaagggcacccgc |
| F125A _ Reverse | gcgggtgcccttggcgttgggcatcggg |
| F125L _ Forward | caacccgatgcccaacttaaagggcaccc |
| F125L _ Reverse | gggtgccctttaagttgggcatcgggttg |
| F125W_ Forward | cccgatgcccaactggaagggcacccgctt |
| F125W_ Reverse | aagcgggtgcccttccagttgggcatcggg |

**SI Table 2.** Mutant primers used in site-directed mutagenesis.


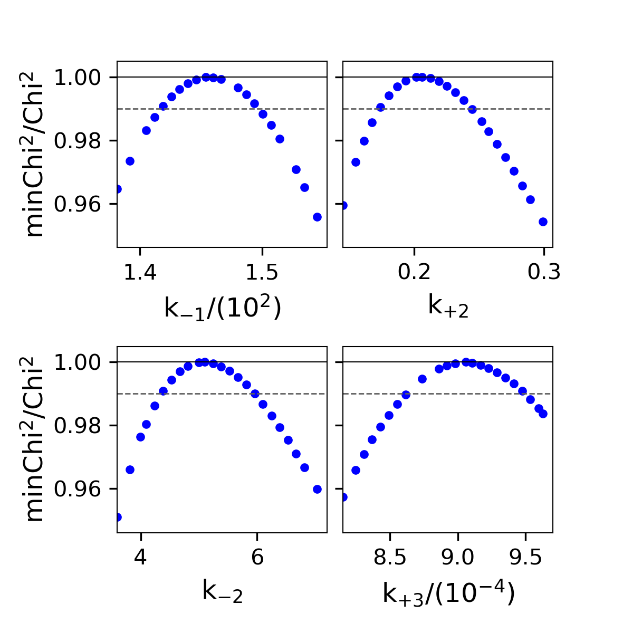

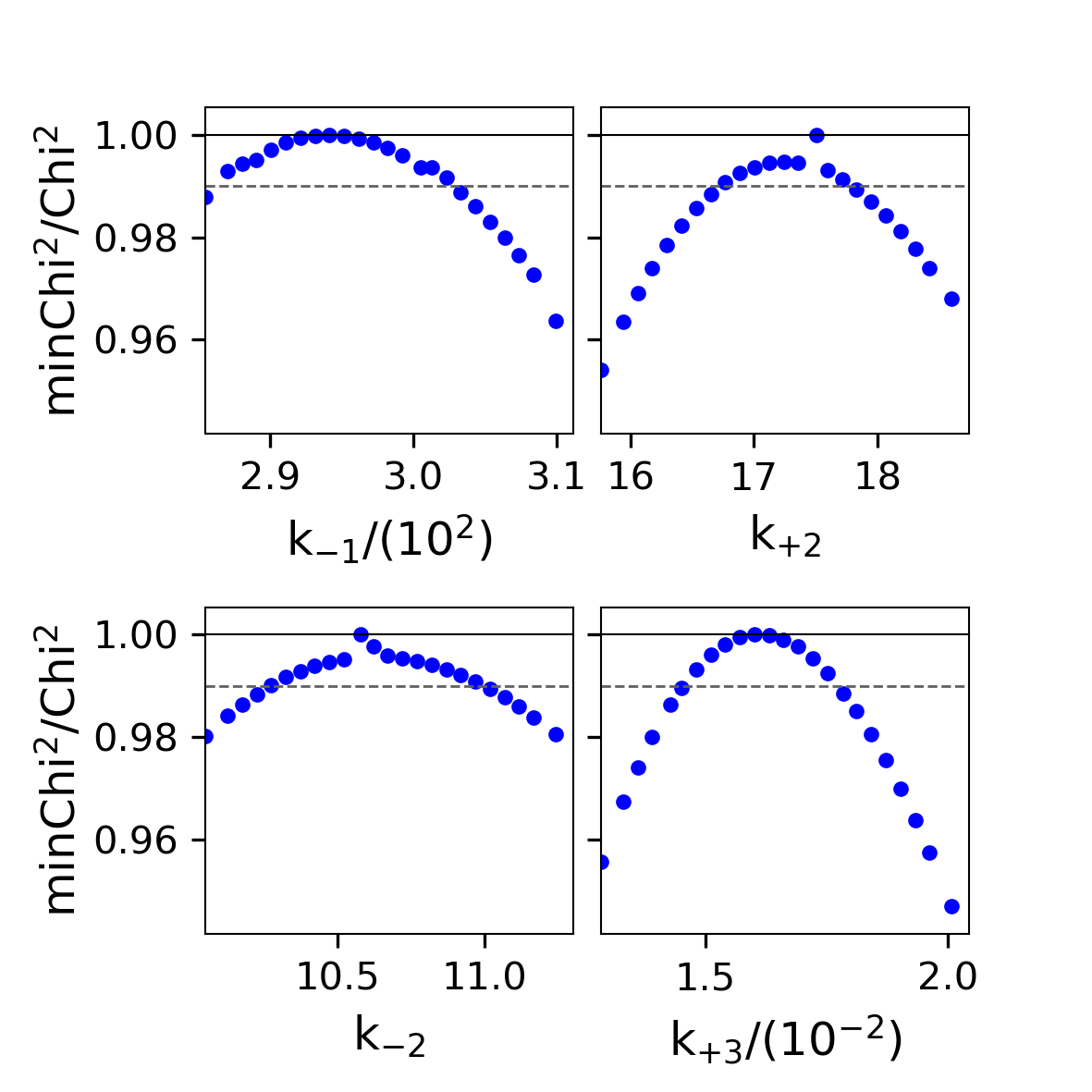

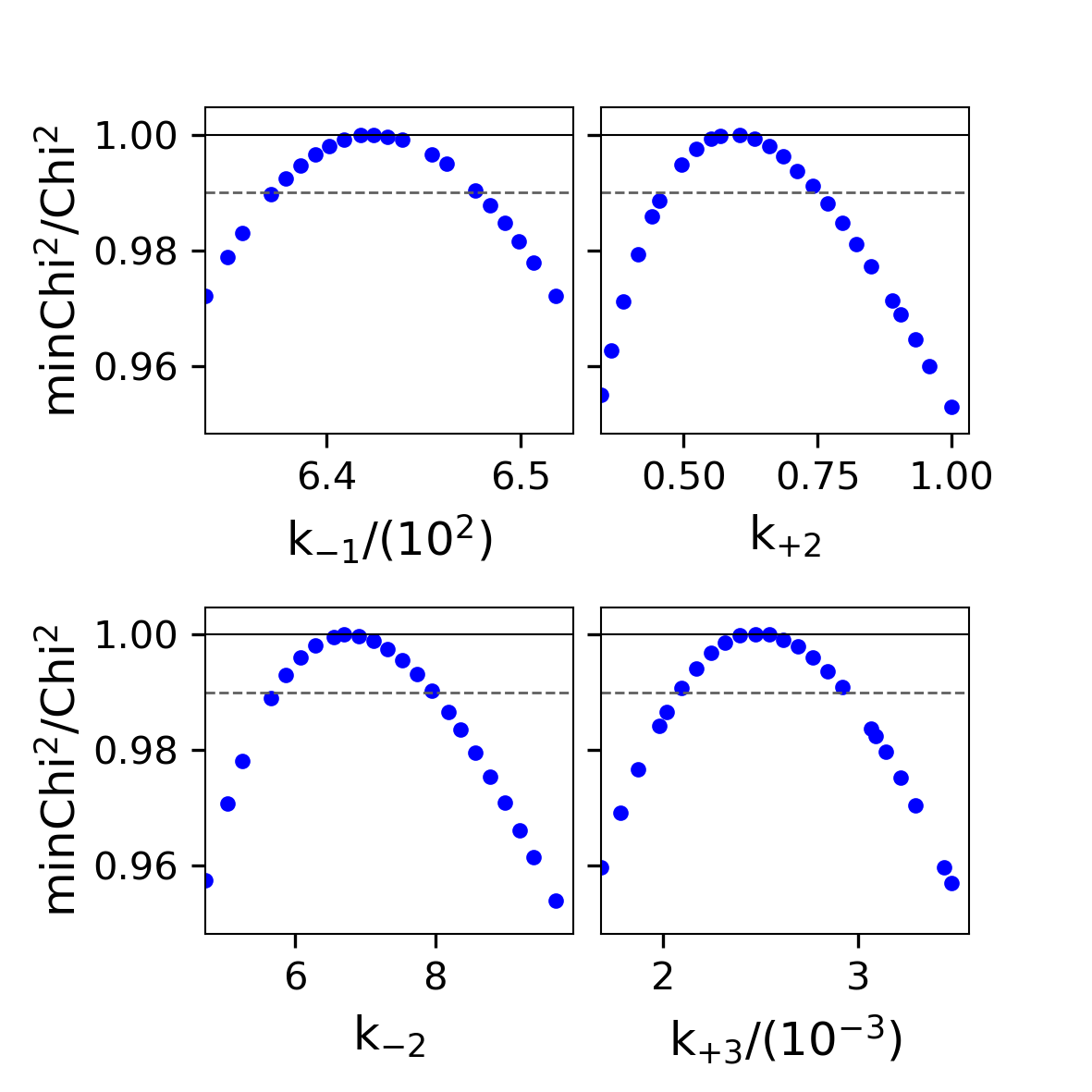

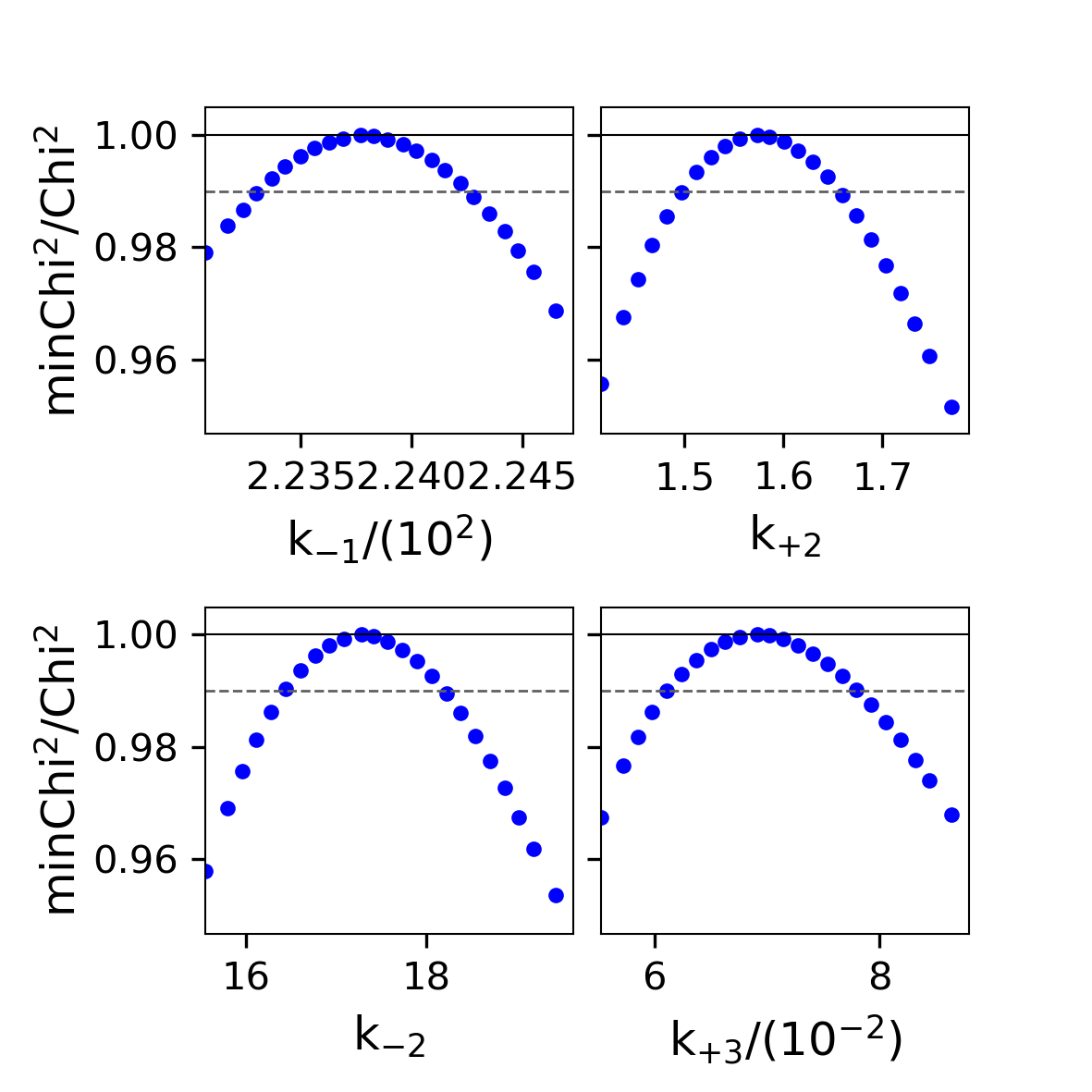


**F125L**

**R129A**

**WT**

**F125A**

**F125W**


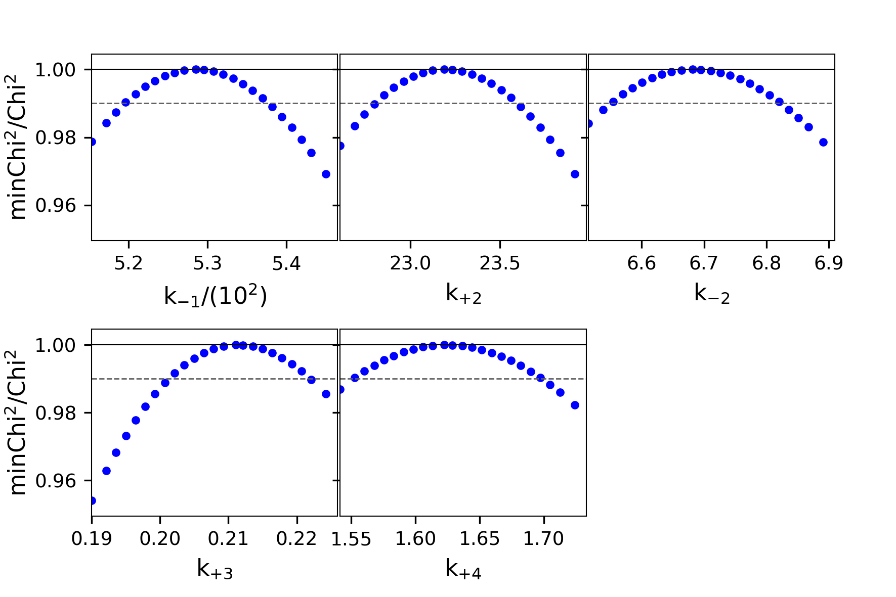

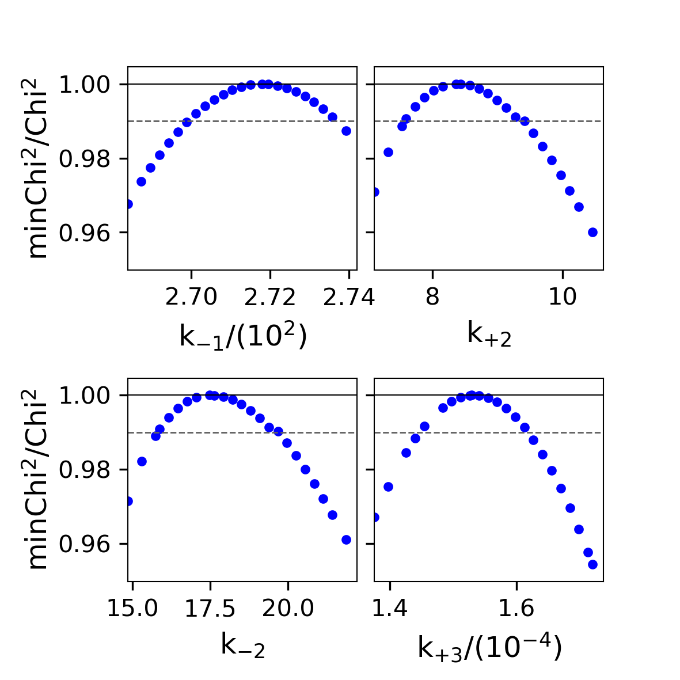

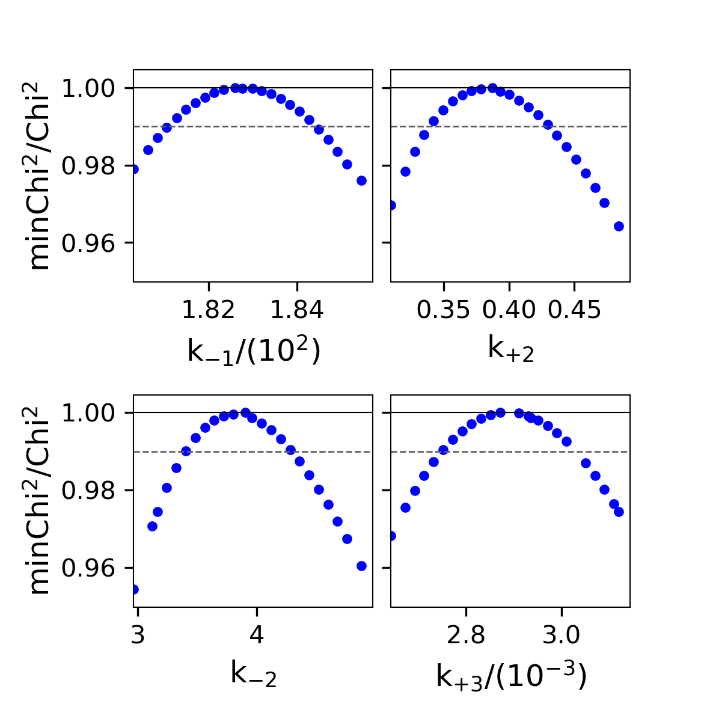


**R44A**

**N124A**

**SI Figure 2. Confidence contour Fitspace analysis from global fitting for WT CcrM and mutants F125L, F125A, F125W, R129A, N124A and R44A.** The data represent the 1D Fitspace calculated for each rate constant. The dashed line establishes the 95% confidence interval at the 0.99 Chi^2^ threshold.

| **Enzyme** | **K_1_** | **K_2_** | ***k_3_*** |
| --- | --- | --- | --- |
| **WT** | 0.19 | 3.5 | 0.21 |
| **F125L** | 0.45 | 0.09 | 0.07 |
| **F125A** | 0.16 | 0.09 | 0.003 |
| **F125W** | 0.33 | 1.65 | 0.02 |
| **R129A** | 0.69 | 0.04 | 0.0009 |
| **N124A** | 0.55 | 0.09 | 0.003 |
| **R44A** | 0.37 | 0.48 | 0.0002 |

**SI Table 3.** Equilibrium constants were derived from the rate constants in Table 1. Since the chemical step was modeled as irreversible, *k_3_* here is not an equilibrium constant but is included for comparison.
